# USP17 is required for peripheral trafficking of lysosomes

**DOI:** 10.15252/embr.202051932

**Published:** 2022-01-26

**Authors:** Jia Lin, Aidan P McCann, Naphannop Sereesongsaeng, Jonathan M Burden, Alhareth A Alsa’d, Roberta E Burden, Ileana Micu, Richard Williams, Sandra Van Schaeybroeck, Emma Evergren, Paul Mullan, Jeremy C Simpson, Christopher J Scott, James F Burrows

**Affiliations:** ^1^ School of Pharmacy Queen’s University Belfast Belfast UK; ^2^ Advanced Imaging Core Technology Unit Faculty of Medicine, Health and Life Sciences Queen’s University Belfast Belfast UK; ^3^ Patrick G Johnston Centre for Cancer Research School of Medicine Dentistry and Biomedical Sciences Queen’s University Belfast Belfast UK; ^4^ School of Biology and Environmental Science UCD Conway Institute of Biomolecular and Biomedical Research University College Dublin Dublin 4 Ireland

**Keywords:** EGF, exocytosis, lysosome, USP17, Membranes & Trafficking, Organelles, Post-translational Modifications & Proteolysis

## Abstract

Expression of the deubiquitinase USP17 is induced by multiple stimuli, including cytokines (IL‐4/6), chemokines (IL‐8, SDF1), and growth factors (EGF), and several studies indicate it is required for cell proliferation and migration. However, the mechanisms via which USP17 impacts upon these cellular functions are unclear. Here, we demonstrate that USP17 depletion prevents peripheral lysosome positioning, as well as trafficking of lysosomes to the cell periphery in response to EGF stimulation. Overexpression of USP17 also increases secretion of the lysosomal protease cathepsin D. In addition, USP17 depletion impairs plasma membrane repair in cells treated with the pore‐forming toxin streptolysin O, further indicating that USP17 is required for lysosome trafficking to the plasma membrane. Finally, we demonstrate that USP17 can deubiquitinate p62, and we propose that USP17 can facilitate peripheral lysosome trafficking by opposing the E3 ligase RNF26 to untether lysosomes from the ER and facilitate lysosome peripheral trafficking, lysosome protease secretion, and plasma membrane repair.

## Graphical abstract

USP17 is required for peripheral lysosome positioning and trafficking in response to EGF stimulation as well as plasma membrane repair.

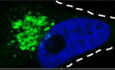

## Introduction

Lysosomes are no longer seen as just static “waste bags” which degrade unwanted proteins, or organelles, brought to them via endocytosis or autophagosomes. Instead, they are recognized as dynamic organelles which traffic throughout the cell and participate in important cellular processes such as nutrient sensing, autophagy, secretion, and plasma membrane repair (Samie & Xu, 2014). Indeed, lysosomal‐related functions such as secretion and plasma membrane repair require the lysosomes to relocate from the “perinuclear cloud” to the plasma membrane. These lysosomes can then fuse with the plasma membrane to release their contents into the extracellular space (Samie & Xu, 2014).

The importance of lysosomes is further emphasized by accumulating evidence that lysosome dysfunction is associated with pathologies including neurodegenerative (Alzheimer’s and Parkinson’s disease) and autoimmune (rheumatoid arthritis and lupus) diseases, as well as cancer (Bonam *et al*, 2019), and upregulation of lysosomal proteins is a conserved feature of aging (Cellerino & Ori, 2017). As a result, it is important that we understand how lysosome positioning is regulated, and what consequences this has on lysosome function.

The DUB/ubiquitin‐specific protease 17 (USP17) family of deubiquitinases was originally identified in mice (DUB‐1, DUB‐1A, and DUB‐2) (Zhu *et al*, 1996, 1997), and the human homolog, USP17/DUB‐3/Dub3 (subsequently referred to as USP17), is induced in response to cytokine, chemokine, and epidermal growth factor (EGF) stimulation (Burrows *et al*, 2004; de la Vega *et al*, 2011; Jaworski *et al*, 2014). USP17 expression is required for proper G1 to S cell cycle progression (McFarlane *et al*, 2010), chemokine‐driven (IL‐8, SDF1) cell motility (de la Vega *et al*, 2011), and EGF receptor (EGFR) clathrin‐mediated endocytosis (Jaworski *et al*, 2014). In addition, we and others have shown that USP17 is overexpressed in a range of tumors when compared to normal tissue (non‐small cell lung cancer (NSCLC), ovarian, breast, colon, esophagus, cervical, and osteosarcoma) (McFarlane *et al*, 2010, 2013; Pereg *et al*, 2010; Zhou *et al*, 2015; Zhang *et al*, 2016; Song *et al*, 2017).

In this study, we show that USP17 is required for peripheral lysosome trafficking, as well as EGF triggered lysosome exocytosis. In addition, we demonstrate that, in the absence of USP17, the repair of plasma membrane damage is dramatically impeded, again indicating USP17 promotes peripheral lysosome trafficking, thus facilitating plasma membrane repair. Finally, we show that USP17 counteracts the impact of the E3 ligase RNF26 on lysosome trafficking by deubiquitinating its substrate p62, indicating it triggers peripheral lysosome trafficking by untethering them from the endoplasmic reticulum (ER).

## Results and Discussion

### USP17 is required for peripheral trafficking of lysosomes

A link between USP17 and the lysosome was previously suggested in two studies indicating USP17 deubiquitinates legumain to regulate its stability (Lin *et al*, 2014; Chen *et al*, 2019). However, we were intrigued how USP17, which we have previously shown to display a perinuclear distribution in HeLa cells (McFarlane *et al*, 2010), and to colocalize with the ER marker calnexin (Burrows *et al*, 2009), could directly impact a lysosomal protease and decided to explore if USP17 could instead impact upon the lysosome.

**Figure EV1:**
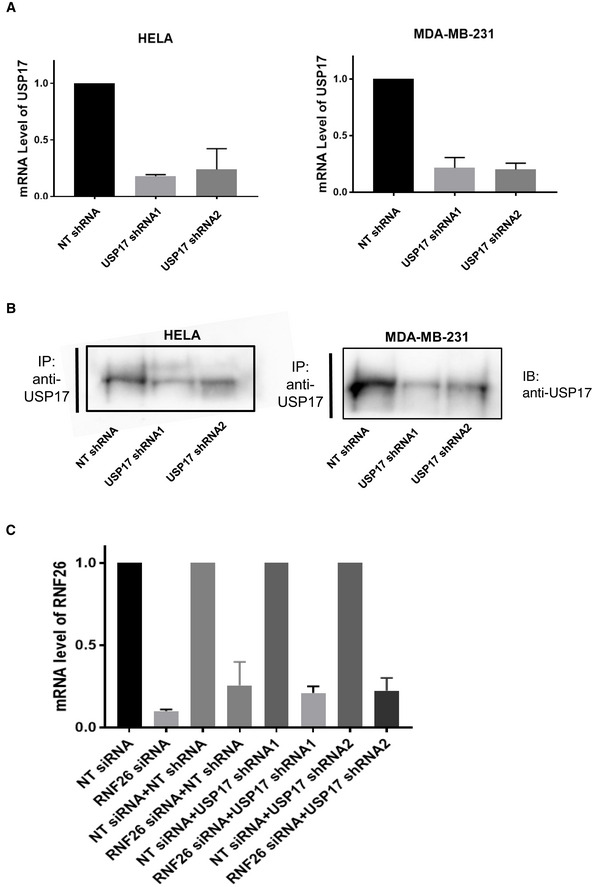
USP17 shRNAs and RNF26 siRNAs deplete USP17 and RNF26 mRNA levels efficiently HeLa and MDA‐MB‐231 cells were transfected with constructs for non‐targeting shRNA, USP17 shRNA1, or USP17 shRNA2. QPCR was carried out to determine relative abundance of USP17 mRNA. The results plotted are representative of results obtained in three separate experiments.HeLa and MDA‐MB‐231 cells were transfected as in A. After 72 h, transfected cells were treated with Bortezomib (100 nM) for 6 h. Cell lysates were prepared and equal amounts of lysate were immunoprecipitated using anti‐USP17 antibody. Immunoprecipitations were immunoblotted with anti‐USP17 antibody, to confirm USP17 depletion.HeLa cells were transfected as in A, in conjunction with non‐targeting control siRNA and RNF26 siRNA as indicated. QPCR was carried out to determine relative abundance of RNF26 mRNA. The results plotted are representative of results obtained in three separate experiments. Data information: Total RNA was extracted for cDNA synthesis and real‐time PCR analysis used the cycle threshold (2−ΔΔCT) method (n = 3). Error bars represent standard errors.

**Figure 1:**
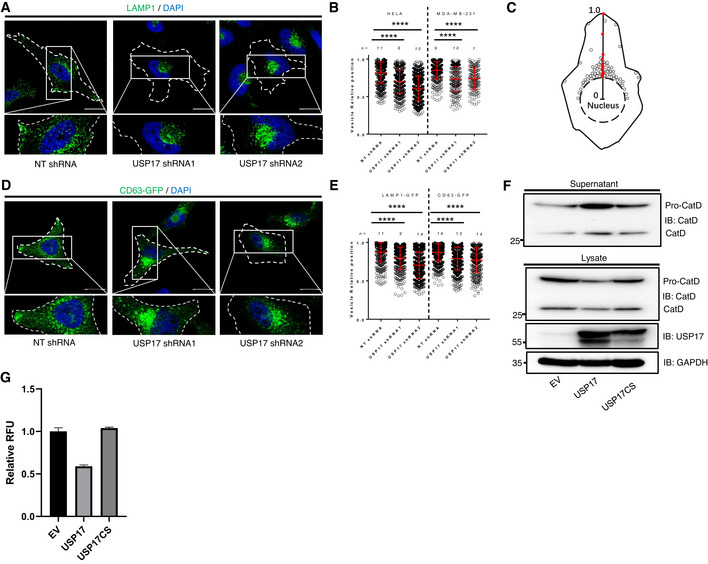
USP17 is necessary for peripheral lysosome trafficking HeLa cells were transfected with constructs for a non‐targeting shRNA (NT), USP17 shRNA1, or USP17 shRNA2. Cells were stained using an anti‐LAMP1 antibody (green) and the nuclei counterstained with DAPI (blue). Lower panels are enlarged images of indicated area in top panels and the cell membrane is marked by dotted line.HeLa and MDA‐MB‐231 cells were transfected as in A and the distribution of LAMP1‐positive vesicles was plotted as vesicle relative position (see C).Vesicle relative position is the ratio between vesicle distance from the nucleus border and the sum of the vesicle’s distances from the nucleus and cell border. If the vesicle is located within the nucleus, the value is set to 0. If the value is 1, then the vesicle is located at the border of cell.HeLa cells transfected as in A, in conjunction with a construct coding GFP‐tagged CD63. The lower panels are enlarged images of indicated area in top panels and the cell membrane is marked by dotted line.HeLa cells transfected as in A, in conjunction with constructs for either GFP‐tagged LAMP1 or CD63. The distribution of GFP‐positive vesicles plotted as vesicle relative position.HeLa cells were transfected with empty vector, or expression constructs for USP17 or USP17CS (inactive mutant). Forty‐eight hours post‐transfection, lysates and cell growth media were harvested and immunoblotted for GAPDH, USP17, and CatD, as indicated.HeLa cells were transfected as in 1F. Forty‐eight hours post‐transfection, lysates were harvested and 5 µg of protein was used in a CatD/E activity assay. Relative fluorescence units (RFU) of triplicate samples at 60 min compared to empty vector control are plotted. The results plotted are representative of results obtained in three separate experiments. Data information: In (A, C), scale bars 25 µm. In (B, D), vesicle position of at least 350 vesicles from a number of cells (n) from a series of confocal images across three separate experiments was analyzed using the IMARIS software package and the distribution of vesicles plotted as vesicle relative position (mean value shown as red bar). Error bars represent standard error; and **** indicates a *P*‐value < 0.0001. One‐way ANOVA was used to determine statistically significant differences between groups.

**Figure EV2:**
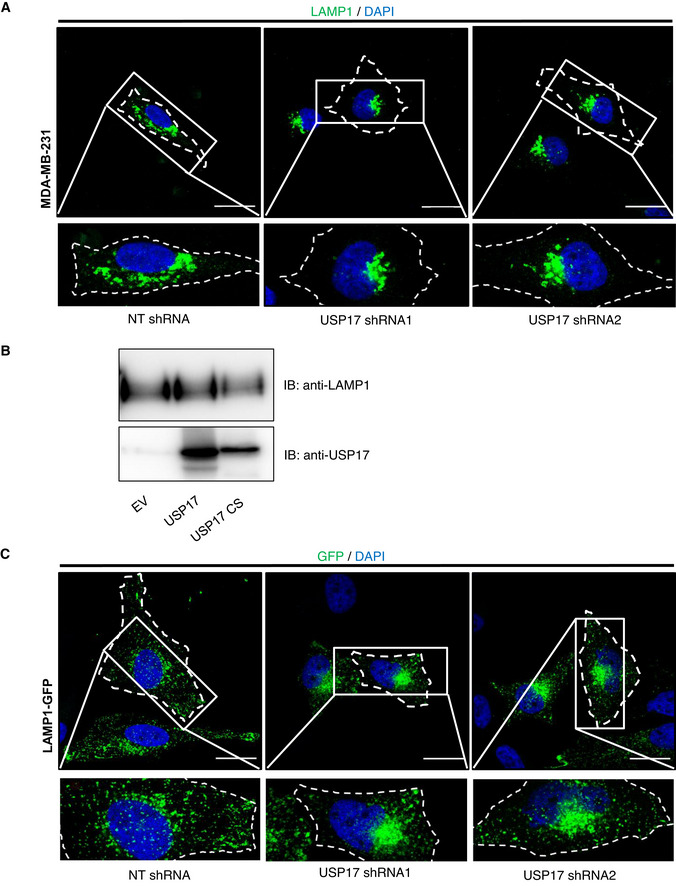
USP17 is necessary for peripheral lysosome trafficking MDA‐MB‐231 cells were transfected with constructs for non‐targeting (NT) shRNA, USP17 shRNA1, or USP17 shRNA2. Seventy‐two hours post‐transfection, the cells were stained for LAMP1 and the nuclei counterstained with DAPI.HeLa cells were transfected with empty vector, or expression constructs for USP17, or USP17CS (inactive mutant). Forty‐eight hours post‐transfection, lysates were harvested and immunoblotted for USP17, and LAMP1, as indicated.HeLa cells were transfected as in A in conjunction with a construct for GFP‐tagged LAMP1. Seventy‐two hours post‐transfection, the nuclei were counterstained with DAPI. Data information: In A and C, lower panels are enlarged images of the indicated area in the top panels and the cell membrane is marked by dotted line. Scale bars 25 µm.

To investigate if USP17 impacts upon lysosome trafficking, we transfected HeLa cells with expression vectors for non‐targeting (NT), or USP17‐specific shRNAs, and knockdown was confirmed by QPCR and Western blotting (Fig EV1A and B). We then assessed the localization of lysosomes via LAMP1 staining using confocal microscopy (Fig 1A and B). In control cells, lysosomes were observed both in the perinuclear region, as well as the periphery of the cell (Fig 1A, left panels). However, USP17 depletion resulted in the lysosomes shifting to a predominantly perinuclear localization (Fig 1A, middle and right panels). Indeed, when we quantified the relative position of the lysosomes to the nucleus (Fig 1C) across multiple cells, there was a significant shift toward the nucleus upon USP17 depletion (Fig 1B, left panel). A similar impact was observed upon the position of LAMP1‐positive vesicles in MDA‐MB‐231 cells transfected as above (Fig EV2A), and again USP17 depletion caused a significant shift of lysosomes toward the nucleus in these cells (Fig 1B, right panel), demonstrating this was not specific to HeLa cells. We also confirmed that the observed change in LAMP1 distribution was not the result of a change in LAMP1 levels, as overexpression of active USP17 did not markedly alter LAMP1 protein levels (Fig EV2B).

We further confirmed this impact upon the lysosome by repeating these experiments in HeLa cells cotransfected with an expression construct for CD63‐GFP, another marker of late endosomes (LEs)/lysosomes. Again, we assessed the localization of CD63‐GFP via confocal microscopy (Fig 1D). In control cells, the CD63‐positive LEs/lysosomes were again observed in the perinuclear region and the periphery of the cell (Fig 1D, left panels). As before, USP17 depletion shifted these LEs/lysosomes to a predominantly perinuclear localization (Fig 1D, middle and right panels). In addition, we cotransfected HeLa cells with an expression construct for LAMP1‐GFP (Fig EV2C), and examined the relative position of both LAMP1‐GFP‐ and CD63‐GFP‐positive vesicles, and as before they both exhibited a significant shift toward the nucleus upon USP17 depletion (Fig 1E).

**Figure EV3:**
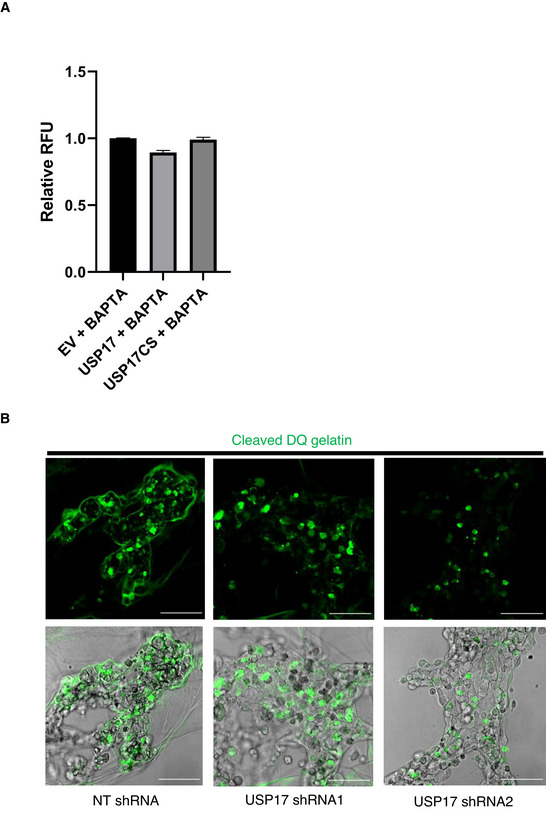
USP17 expression triggers secretion of lysosomal proteases HeLa cells were transfected with empty vector, or expression constructs for USP17, or USP17CS (inactive mutant), as indicated. Cells were treated for 3 h with BAPTA‐AM (30 µM) prior to making lysates. Forty‐eight hours post‐transfection, lysates were harvested and 5 µg of protein used in a CatD/E activity assay. RFU of triplicate samples at 60 min compared to empty vector control are plotted. The results plotted are representative of results obtained in three separate experiments. Error bars represent standard error.HeLa cells were transfected with constructs for non‐targeting (NT) shRNA, USP17 shRNA1, or USP17 shRNA2. Seventy‐two hours post‐transfection, the cells were incubated in a DQ‐gelatin/matrigel solution (25 µg/ml) to compare the proteolytic ability of the transfected cells. Proteolysis was then assessed in fluorescent (top panel) and bright‐field and fluorescent images (bottom panel) taken using confocal microscopy. Scale bars 100 µm.

To further confirm this impact upon the lysosome, we transfected HeLa cells with either an empty vector, or expression plasmids for USP17 or USP17CS (inactive mutant), and examined endogenous levels of the abundant lysosomal protease cathepsin D (CatD). As had been observed for legumain (Lin *et al*, 2014; Chen *et al*, 2019), overexpression of active USP17 results in a drop in intracellular CatD (Fig 1F, bottom panels), again indicating an impact upon the lysosome, rather than just legumain. We further confirmed this drop in intracellular CatD via a fluorescent protease activity assay using a CatD/E‐specific substrate. Overexpression of active USP17, but not USP17CS, resulted in a drop in CatD/E activity in lysates taken from these cells (Fig 1G). The addition of the intracellular calcium chelator BAPTA‐AM (30 µM for 3 h) blunted the impact of USP17 overexpression upon CatD/E activity, indicating this impact is Ca^2+^ dependent (Fig EV3A). More interestingly, when we examined extracellular levels of CatD, we found that USP17 overexpression triggered increased secretion (Fig 1F, top panel). This indicated USP17, rather than impacting upon protein stability, was triggering the secretion of CatD, by triggering lysosome exocytosis. In addition, the presence of bands representative of pro‐ and mature CatD in the supernatant indicated that mature enzyme is present in the supernatant (Fig 1F, top panel).

To further confirm USP17 can facilitate lysosome exocytosis and the secretion of active lysosomal proteases, we undertook a live cell proteolysis assay to examine the impact of USP17 depletion upon cleavage of DQ gelatin, which we have previously shown to be cleaved by cathepsins (Small *et al*, 2013). The degradation of this substrate in the pericellular environment results in the emission of a bright green fluorescence, which was noticeably decreased in Hela cells transfected with the USP17 shRNAs, when compared to control cells (Fig EV3B). This reinforced the previous observations indicating that USP17 facilitates peripheral trafficking of LEs/lysosomes and its expression can trigger the secretion of lysosomal proteases.

### USP17 is required for EGF‐driven lysosome exocytosis

**Figure 2:**
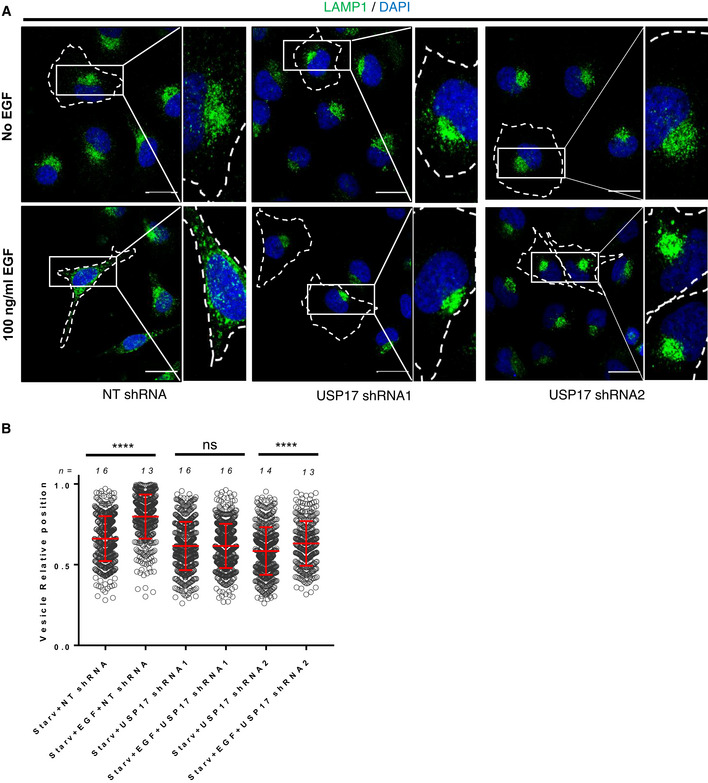
USP17 is necessary for EGF‐mediated peripheral lysosome trafficking HeLa cells were transfected with constructs for a non‐targeting (NT) shRNA, USP17 shRNA1, or USP17 shRNA2. Forty‐eight hours post‐transfection, the cells were either serum starved (upper panels), or treated with serum‐free medium containing 100 µg/ml EGF (lower panels) for 16 h prior to staining for LAMP1 (green) and DAPI (blue). Right hand panels are enlarged images of the indicated area in left panels and the cell membrane is marked by dotted line. Scale bars 25 μm.The distribution of at least 300 LAMP1‐positive vesicles from a number of cells (n) from a series of confocal images across three separate experiments was plotted as vesicle relative position (mean value is red bar). Error bars represent standard error, ns indicates not significant, and **** indicates *P*‐values < 0.0001. One‐way ANOVA was used to determine statistically significant differences between groups.

**Figure EV4:**
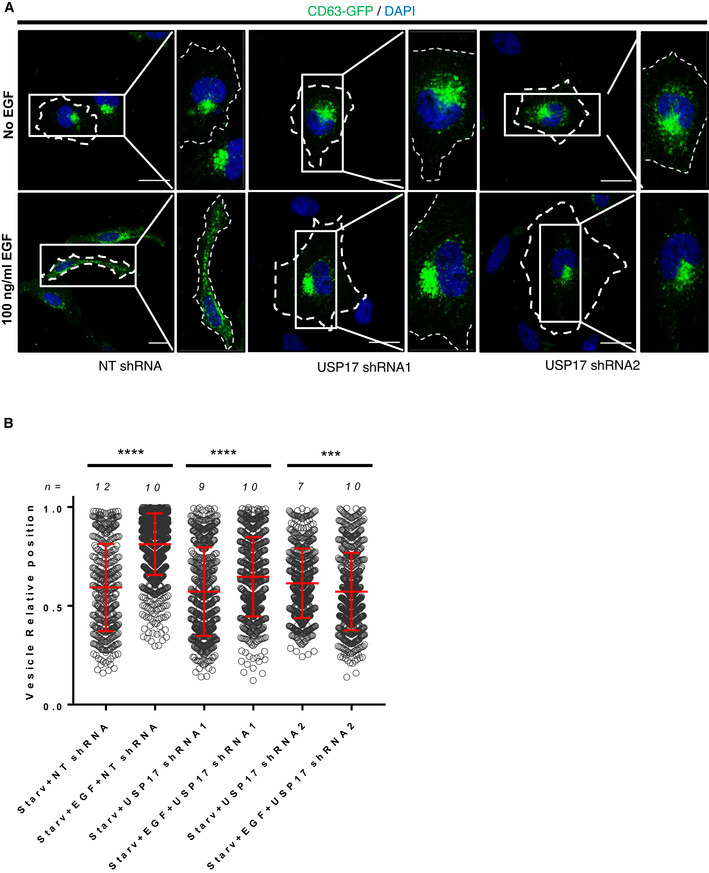
USP17 is necessary for EGF‐mediated peripheral lysosome trafficking HeLa cells were transfected with constructs coding a non‐targeting (NT) shRNA, USP17 shRNA1, or USP17 shRNA2 in conjunction with GFP‐tagged CD63 as indicated. Forty‐eight hours post‐transfection, the cells were either serum starved (upper panels), or placed in serum‐free medium with 100 µg/ml EGF (lower panels) for 16 h prior to being stained with DAPI. Right hand panels are enlarged images of the indicated area in left panels and the cell membrane is marked by dotted line.The distribution of at least 400 GFP‐positive vesicles from a number of cells (n) from a series of confocal images across three separate experiments was plotted as vesicle relative position (mean value is red bar). Error bars represent standard error and **** indicates *P*‐values < 0.0001. One‐way ANOVA was used to determine statistically significant differences between groups.

USP17 is induced by multiple stimuli including EGF (Burrows *et al*, 2004; de la Vega *et al*, 2011; Jaworski *et al*, 2014), and we have shown that USP17 expression is required for cell migration triggered by several of these stimuli (de la Vega *et al*, 2011). Interestingly, EGF triggers peripheral lysosome trafficking, something which is required for EGF‐driven cell invasion and motility (Dykes *et al*, 2017). This suggested USP17 induction by these stimuli could be driving peripheral trafficking of lysosomes to allow cell migration. To confirm this, HeLa cells were transfected with non‐targeting (NT), or USP17‐specific shRNAs, as indicated, and the cells were either starved or stimulated with EGF, as had been done before (Dykes *et al*, 2017). We then examined the localization of the lysosome by assessing LAMP1 staining as above (Fig 2A). In the control cells, starvation caused perinuclear accumulation of the lysosomes, and they were redistributed throughout the cell upon EGF treatment (Fig 2A, left panels). However, in the USP17‐depleted cells, EGF treatment had little or no impact upon lysosome localization (Fig 2A, middle and left panels). This was confirmed by measuring the relative lysosome position across a number of cells in each condition (Fig 2B), which revealed EGF caused a significant peripheral shift in lysosome localization in control cells, but this was severely blunted in USP17‐depleted cells (Fig 2B). We further confirmed these observations using HeLa cells cotransfected with CD63‐GFP, and again USP17 depletion prevented EGF‐driven peripheral trafficking of the CD63‐positive vesicles (Fig EV4A and B).

Together, these data indicate USP17 expression is required for LEs/lysosome peripheral trafficking in response to EGF, something which fits with the previous observations that EGF requires lysosome exocytosis to promote cell migration (Dykes *et al*, 2017), that USP17 is induced by EGF (Jaworski *et al*, 2014), and USP17 induction is required for cell migration (de la Vega *et al*, 2011).

### USP17 is required to facilitate plasma membrane repair

**Figure 3:**
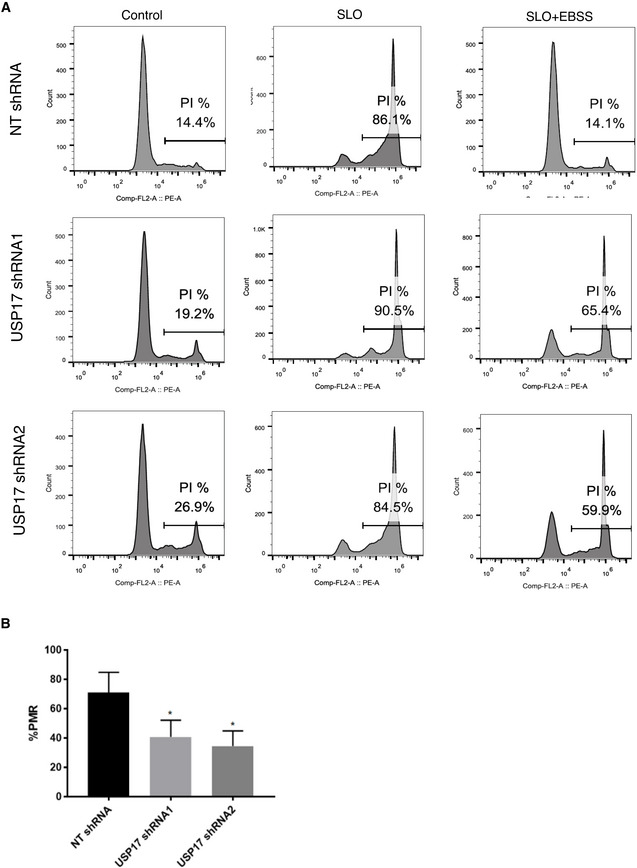
USP17 is required for plasma membrane repair HeLa cells were transfected with constructs encoding a non‐targeting (NT) shRNA, USP17 shRNA1, or USP17 shRNA2, as indicated.
Forty‐eight hours post‐transfection, the cells were washed with Ca^2+^ and Mg^2+^ free ice‐cold HBSS. The different cell populations were split into three aliquots. One aliquot was placed into fresh medium (left panels). SLO was added to other two aliquots and then the toxin‐containing medium was removed and the aliquots were either placed into Ca^2+^ and Mg^2+^ free HBSS (Middle panels), or EBSS containing Ca^2+^ and Mg^2+^ (right panels) prior to staining with propidium iodide and analysis by flow cytometry.The percentage plasma membrane repair (PMR) was calculated according to the following formula: 1 − (% PI‐positive cells with Ca^2+^/% PI‐positive cells without Ca^2+^) × 100. Three separate experiments were analyzed and plotted. Error bars represent standard error, and * indicates *P*‐values < 0.05. One‐way ANOVA was used to determine statistically significant differences between groups.

Plasma membrane damage is a frequent event in mammalian cells, especially in cells under mechanical stress, or under attack from invading pathogens which utilize pore‐forming toxins, and restoring membrane integrity is essential for cell survival (Andrews & Perez, 2015). Several mechanisms have been proposed to facilitate plasma membrane repair, one of which is the fusion of lysosomes with the plasma membrane triggered by a damage‐induced calcium influx (Reddy *et al*, 2001). This allows the lysosomal enzyme acid sphingomyelinase to be secreted causing the production of ceramide at the plasma membrane, triggering endocytosis of damaged membrane and restoring plasma membrane integrity (Tam *et al*, 2010). In addition, secretion of the lysosomal proteases cathepsins B, D, and L has also been shown to contribute to the regulation of this process (Castro‐Gomes *et al*, 2016). As we had found USP17 to facilitate peripheral trafficking of LEs/lysosomes, and the secretion of lysosomal proteases, we hypothesized that USP17 would also facilitate proper plasma membrane repair as an important mechanism of plasma membrane repair requires lysosome exocytosis (Reddy *et al*, 2001). To assess this, we transfected HeLa cells as before with the non‐targeting and USP17‐specific shRNAs and triggered plasma membrane damage using streptolysin O (SLO), a bacterial protein that forms pores in the plasma membrane and is routinely used to assess plasma membrane repair (Andrews & Perez, 2015; Encarnação *et al*, 2016). The cells were initially incubated with SLO in the absence of Ca^2+^ to prevent repair being initiated, as Ca^2+^ is required to trigger lysosomal exocytosis and fusion with the plasma membrane. The cells then either remained in the absence of Ca^2+^ (Fig 3A, middle panels), or were transferred to EBSS buffer containing Ca^2+^ to allow plasma membrane repair to proceed (Fig 3A, right panels). The cells were then incubated with propidium iodide (PI) and uptake assessed via flow cytometry (Fig 3A). In all cases, some cells in each population exhibited damage prior to SLO treatment due to the observation of PI uptake (Fig 3A, left panels). However, SLO treatment dramatically increased the percentage of cells displaying plasma membrane damage, with over 80% of the cells assayed showing PI uptake in all populations when these pores were not repaired in the absence of Ca^2+^ (Fig 3A, middle panels). However, upon the introduction of Ca^2+^ via EBSS buffer, the control cells reduced PI uptake back to basal levels (Fig 3A, top right panel), indicating the majority of pores triggered by SLO treatment were repaired and that plasma membrane repair was functioning correctly. In contrast, in cells depleted of USP17, the levels of PI uptake remained at two to three times the basal rate even when they were exposed to Ca^2+^ (Fig 3A, middle and bottom right panels), suggesting plasma membrane repair was not functioning correctly in the absence of USP17. As done previously (Encarnação *et al*, 2016), we then calculated the percentage plasma membrane repair and observed that this dropped significantly upon USP17 depletion when compared to control cells (Fig 3B). This all indicated that the loss of peripheral lysosomes in the absence of USP17 impairs the ability of these cells to fully repair their plasma membrane when damaged by SLO, and further supported the data indicating that USP17 is required for lysosome exocytosis.

### USP17 deubiquitylates p62 and opposes RNF26 to allow lysosome exocytosis

**Figure 4:**
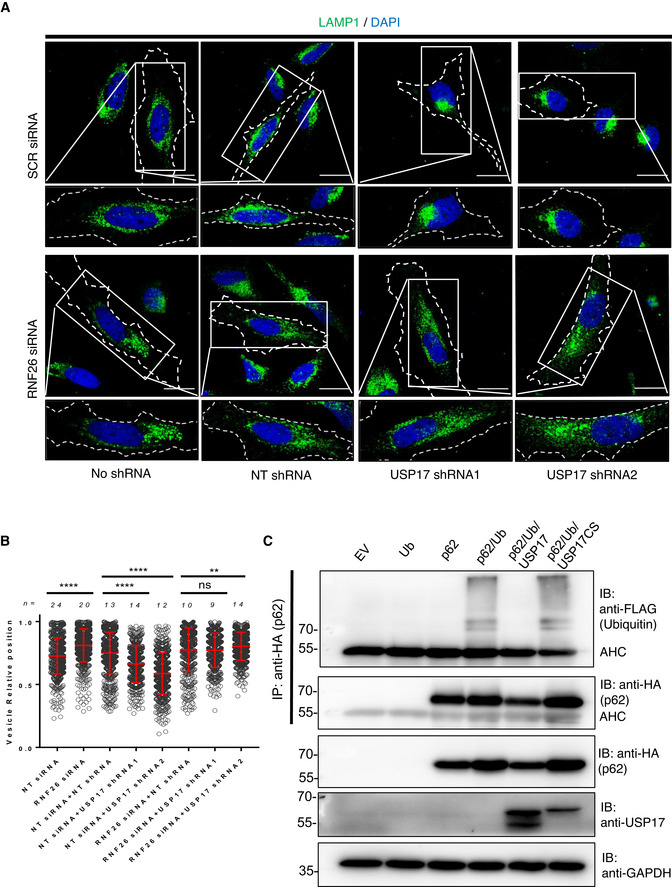
USP17 opposes the action of RNF26 and deubiquitylates p62 HeLa cells were transfected with negative control siRNA and RNF26 siRNA as well as constructs coding a non‐targeting (NT) shRNA, USP17 shRNA1, or USP17 shRNA2, as indicated. Forty‐eight hours post‐transfection, cells were stained for LAMP‐1 (green) and DAPI (blue). Lower panels are enlarged images of indicated area in top panels and the cell membrane is marked by dotted line. Scale bars 25 μm.The distribution of at least 400 LAMP1‐positive vesicles from a number of cells (n) from a series of confocal images across three separate experiments was plotted as vesicle relative position (mean value is red bar). Error bars represent standard error, ns indicates not significant, ** indicates *P*‐values < 0.01, and **** indicates *P*‐values < 0.0001. One‐way ANOVA was used to determine statistically significant differences between groups.HeLa cells were transfected with expression constructs for HA‐tagged p62 and FLAG‐tagged ubiquitin as well as empty vector, USP17, or USP17CS. After 48 h, cell lysates were prepared and HA‐tagged proteins were pulled down using anti‐HA agarose. Pull downs and lysates were immunoblotted with anti‐HA, anti‐FLAG, and anti‐USP17 antibodies, as indicated, to confirm the presence of ubiquitinated p62.

Lysosome positioning and trafficking is regulated in multiple ways, but the previous localization of USP17 to the ER led us to hypothesize that USP17 regulates lysosome tethering to the ER, something which has previously been demonstrated to be facilitated via a number of mechanisms (Bonifacino & Neefjes, 2017). One mechanism of lysosomal tethering to the ER is via the E3 ligase RNF26, which ubiquitinates p62 allowing it to act as a bridge between the ER and lysosomes, and tethers them in the perinuclear cloud (Jongsma *et al*, 2016; Bonifacino & Neefjes, 2017). Therefore, we examined if USP17 could reverse the impact of RNF26, as this was the only tethering mechanism known to involve ubiquitin. To determine if USP17 opposes the action of RNF26, we again depleted USP17 in combination with RNF26‐specific and control siRNAs (Fig EV1C) and then examined LAMP1 localization (Fig 4A). As expected, when RNF26 was depleted using the siRNA, this resulted in the LAMP1‐positive lysosomes being distributed more toward the periphery of the cell (Fig 4A, left panels, Fig 4B, lanes 1–2). The addition of the non‐targeting control shRNA made little difference to the distribution of the lysosomes in the presence or absence of RNF26 (Fig 4A, middle left panels, Fig 4B, lanes 3, 6). In the control cells, USP17 depletion again caused perinuclear accumulation of the lysosomes (Fig 4A, top right and top middle right panels). However, when RNF26 was also depleted, the lysosomes were distributed more toward the periphery of the cell, and USP17 depletion did not result in perinuclear lysosome accumulation (Fig 4A, bottom middle right and bottom right panels). Indeed, when the relative position of the lysosomes was assessed, USP17 depletion again resulted in a significant shift toward the nucleus in control cells (Fig 4B, lanes 4‐5), but in cells which had also been depleted of RNF26, the impact of USP17 depletion was less significant (Fig 4B, lanes 7–8), and where it had an impact it did the opposite, spreading the lysosomes to the periphery (Fig 4B, lane 8).

**Figure EV5:**
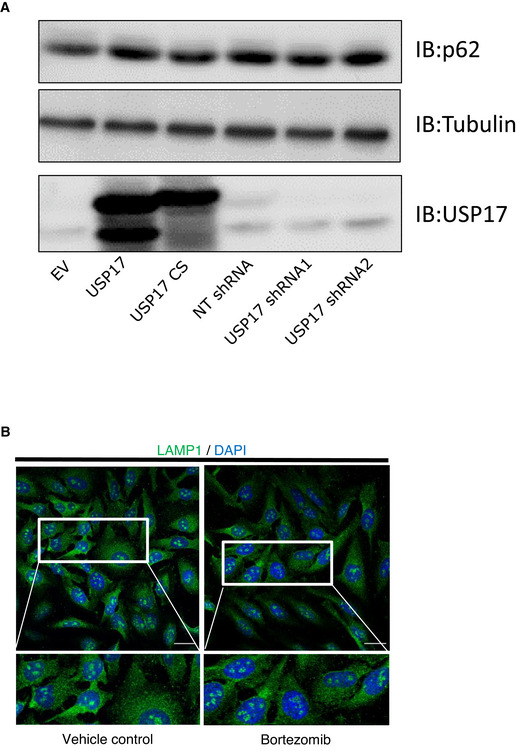
USP17 does not impact upon p62 levels via the proteasome HeLa cells were transfected with empty vector, or expression constructs for USP17 and USP17CS (inactive mutant), or coding a non‐targeting (NT) shRNA, USP17 shRNA1, or USP17 shRNA2, as indicated. Forty‐eight hours post‐transfection, lysates were harvested and immunoblotted for tubulin, USP17, and p62, as indicated.HeLa cells were treated with vehicle control, or Bortezomib (100 nM), as indicated. After 6 h, cells were stained for LAMP‐1 (green) and DAPI (blue). Lower panels are enlarged images of indicated area in top panels. Scale bars 25 µm.

This indicated USP17 opposed the action of RNF26, and therefore we examined USP17s impact upon p62 ubiquitination. We transfected HeLa cells with the indicated constructs expressing HA‐tagged p62, FLAG‐tagged ubiquitin, and USP17 or USP17CS, and did pull downs using anti‐HA agarose. USP17 expression resulted in a loss of the monoubiquitin from p62, something not observed with the inactive mutant (Fig 4C). This indicates USP17 deubiquinates p62 to release lysosomes from the perinuclear cloud, facilitating their peripheral trafficking, exocytosis, and plasma membrane repair. To further confirm that this impact was due to the monoubiquitination and tethering mechanism, we examined the impact of USP17 overexpression and depletion upon endogenous p62 levels, and observed no marked alteration (Fig EV5A). This indicated USP17 was not regulating p62 turnover, via the proteasome, or autophagy. The was supported further by the observation that treating HeLa cells with the proteasome inhibitor Bortezomib had no obvious impact upon LAMP1 localization (Fig EV5B).

**Figure 5:**
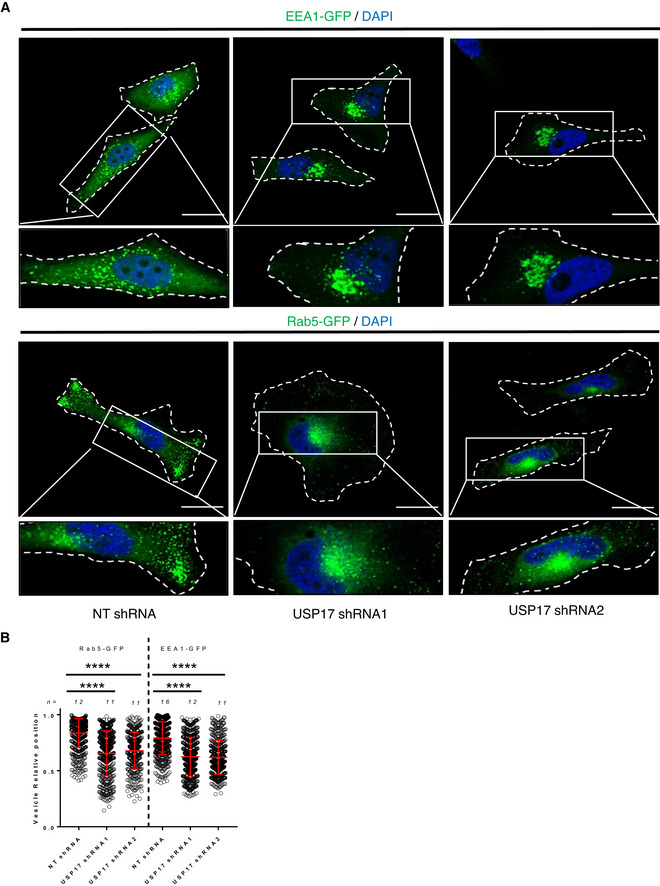
USP17 is necessary for peripheral trafficking of early endosomes HeLa cells were transfected with constructs for EEA1‐GFP and RAB5‐GFP in addition to non‐targeting scrambled (NT) shRNA, USP17 shRNA1, or USP17 shRNA2. Seventy‐two hours post‐transfection, the cells were stained with DAPI. Scale bars 25 μm.The distribution of at least 400 EEA1 or RAB5‐positive vesicles from a number of cells (n) from a series of confocal images across three separate experiments was plotted as vesicle relative position (mean value is red bar). Error bars represent standard error and **** indicates a *P*‐values < 0.0001. One‐way ANOVA was used to determine statistically significant differences between groups.

In the original study examining the impact of RNF26 upon lysosome tethering, it was also found to be involved in tethering other endocytic vesicles to the ER, including early endosomes (Jongsma *et al*, 2016). Therefore, to further confirm that USP17 opposes the action of RNF26, we transfected HeLa cells with non‐targeting (NT) and USP17‐specific shRNAs, as well as expression constructs for RAB5‐GFP or EEA1‐GFP, markers of early endosomes. Both of these markers had been shown to be distributed to the cell periphery upon RNF26 depletion, indicating RNF26 tethers early endosomes to the ER (Jongsma *et al*, 2016). Depletion of USP17 resulted in the clustering of both RAB5 and EEA1‐positive endosomes toward the perinuclear region (Fig 5A), and a significant shift was confirmed by measuring the relative position of individual RAB5 and EEA1‐positive endosomes (Fig 5B). This again indicated USP17 opposes the impact of RNF26 and further reinforces the conclusion that USP17 acts to deubiquitinate p62 and release these vesicles from the perinuclear cloud.

There are a limited number of studies examining RNF26, including two of which indicate RNF26 regulates interferon (IFN) production and STING‐dependent innate immune signaling (Qin *et al*, 2014; Fenech *et al*, 2020). Interestingly, USP17 has previously been shown to be required for RIG‐I/MDA5‐dependent IFN production, indicating USP17 may act to counter RNF26 in more than one context (Chen *et al*, 2010). In addition, RNF26 depletion blocks EGF trafficking to the perinuclear cloud from the cell periphery, as well as EGFR degradation upon EGF engagement, and it redistributes the transferrin receptor (TfR) toward the cell periphery (Jongsma *et al*, 2016). Two subsequent studies have also indicated RNF26 facilitates the targeting of EGFR to the lysosome for degradation (Cremer *et al*, 2021; Torrino *et al*, 2021). We have shown that USP17 is required for clathrin‐mediated endocytosis of EGFR and TfR, and that USP17 depletion also blocks EGFR degradation upon EGF engagement, although it blocks its endocytosis, rather than its subsequent targeting to the lysosome (Jaworski *et al*, 2014). This indicates both RNF26 and USP17 are required to facilitate the trafficking of these receptors, and further reinforces the connection between them.

This molecular mechanism could also potentially explain USP17’s role in cancer. As mentioned before, USP17 is overexpressed in a range of tumors when compared to normal tissue (NSCLC, ovarian, breast, colon, esophagus, cervical, and osteosarcoma) (McFarlane *et al*, 2010, 2013; Pereg *et al*, 2010; Zhou *et al*, 2015; Zhang *et al*, 2016; Song *et al*, 2017), and high USP17 expression has also been associated with recurrence and metastases in NSCLC (McFarlane *et al*, 2013). Interestingly, a study which screened for proteins which could drive lung cancer metastases identified the most potent driver of metastases as TMEM106B, which drives lysosome biogenesis and exocytosis, and indicated lysosome exocytosis was required for its ability to drive metastases (Kundu *et al*, 2018). This could indicate that USP17 overexpression in tumors could drive cell migration by allowing the release of lysosomes to the cell periphery, as seen in response to EGF (Dykes *et al*, 2017), and this could facilitate metastases.

In the original study which identified RNF26 as the ligase targeting p62, USP15 was also identified as a RNF26 binder and found to deubiquitinate p62 (Jongsma *et al*, 2016). Silencing USP15 ablated highly mobile peripheral lysosomes marked by lysotracker and resulted in an overall decrease in lysosome mobility, although it did not significantly alter the distribution of CD63‐positive LEs/lysosomes (Jongsma *et al*, 2016). The data presented here indicate USP17 only has an impact in the presence of RNF26, can deubiquitinate p62, and has a significant impact upon LE/lysosome positioning. This indicates USP17 is able to counteract the ubiquitination of p62 by RNF26, and untether LEs/lysosomes from the ER to allow their peripheral trafficking. However, it is possible that both USP15 and USP17 are required to counteract RNF26 ubiquitination of p62, but under different contexts. In particular, USP17, which is induced by multiple extracellular stimuli (Burrows *et al*, 2004; de la Vega *et al*, 2011; Jaworski *et al*, 2014), may be of particular importance in mediating lysosome peripheral trafficking in response to these extracellular signals, as we have shown for EGF. However, further studies are required to further delineate the relationship among RNF26, USP15, and USP17 to determine in which contexts each is important and how each of them contributes to the various different functions which have been attributed to them individually.

## Materials and Methods

### Plasmids

The pDQ‐EV, pDQ‐USP17, pDQ‐USP17CS, USP17 shRNA1 (pSUPER‐USP17shRNA), USP17 shRNA2 (pRS‐USP17shRNA), and non‐targeting shRNA (pRS‐scrambled shRNA) were previously described (McFarlane *et al*, 2010; de la Vega *et al*, 2011; Jaworski *et al*, 2014). Constructs for HA‐p62/SQSTM1 (Plasmid #28027), CD63‐pEGFP (Plasmid #62964), and Lamp1‐GFP (Plasmid #34831) were obtained from Addgene. RNF26 and NT siRNA were previously described (Jongsma *et al*, 2016).

### Cell culture and DNA transfections

HeLa and MDA‐MB‐231 cells (American Type Culture Collection (ATCC), Manassas, USA) were grown in DMEM supplemented with 10% FCS, 1% penicillin (10,000 U/ml)/streptomycin (10,000 µg/ml), and 1% l‐glutamine (200 mM) (Thermo Fisher Scientific, Waltham, USA). Cells lines were grown at 37°C in a 5% CO_2_ humidified incubator.

Cells were transfected with Lipofectamine 3000 (HeLa; Thermo Fisher Scientific, Waltham, USA) or Xtreme‐GENE HP ^TM^ (MDA‐MB‐231; Roche Diagnostics, Indianapolis, USA) transfection reagents according to manufacturer’s instructions. Cells were seeded between 0.5 × 10^6^ and 1.0 × 10^6^ cells for cell cycle analysis or protein experiments, or 0.7–2.5 × 10^4^ on four‐well glass culture slides (BD Falcon, Bedford, USA) for microscopy experiments. The cells were transfected with 2 µg of plasmid DNA for protein experiments and biological assays, or 0.25 µg of plasmid DNA for confocal microscopy experiments. For those experiments with EGF stimulation, cells were rested in EBSS (Thermo Fisher Scientific, Waltham, USA) medium without serum and stimulated with 100 ng/ml recombinant human EGF (Thermo Fisher Scientific, Waltham, USA) for the indicated times as had been done previously (Dykes *et al*, 2017).

### Confocal microscopy

Cells were seeded at 0.7–2.5 × 10^4^ cells/1.7 cm^2^ well of glass culture slides (BD Falcon, Bedford, USA). Cells were transfected as previously described. The cells were fixed in 4% paraformaldehyde (Sigma‐Aldrich, Steinheim, Germany), in PBS for 20 min. The cells were then permeabilized in 0.5% Triton X‐100 in PBS for 5 min, washed in PBS, and blocked in blocking solution (1% BSA, 10% donkey serum [both from Sigma‐Aldrich, Steinheim, Germany] in PBS) for 1 h at RT. Transfected proteins and cell organelles were stained with appropriate antibodies or counterstains according to manufacturer’s protocol. Antibodies and costains were as follows: mouse anti‐LAMP1 (1:1,000; Abcam, Cambridge, UK; Cat no: ab25630) and donkey anti‐mouse Alexa Fluor 488 (1:200, Thermo Fisher Scientific‐Invitrogen, Waltham, USA; Cat no: A32766). The slides were sealed with a coverslip and Prolong Gold antifade mounting media with DAPI (Thermo Fisher Scientific, Waltham, USA). Fluorescent images were visualized on a Leica TCS SP8 (Leica Microsystems, Milton Keynes, UK) Confocal inverted microscope with an oil immersion objective with 63× magnification and 1.4 NA (Numerical Aperture). Fluorescent images were captured with a 1024 × 1024 frame and 400 Hz scanning speed. Samples were excited with 405 and 488 nm line laser and fluorescence emission was collected using spectral HyD detectors. Images were analyzed using Leica LAS X software and images presented in the same figure were captured using standardized settings and exposure times.

### Relative vesicle position

As lysosomes, late endosomes and early endosomes have vesicular‐like structure, we can approximate clusters of markers of these vesicles such as LAMP1, CD63, RAB5, and EEA1 with vesicles and use tools to analyze their localization within the cell. Relative vesicle position is the ratio between the vesicle distance from the nucleus border and the sum of the vesicle’s distances from the nucleus and cell border. If the vesicle is located within the nucleus, the value is set to 0. If the value is 1, then the vesicle is located at the border of the cell. This was calculated using IMARIS 9.3 software (Bitplane AG, Zurich, Switzerland), setting consistent thresholds for cell diameter and vesicles for all images. Thresholds and other parameters were chosen as appropriate based on control samples within each experiment.

### DQ‐gelatin proteolysis assay

HeLa cells were transfected as outlined. Transfected cells were trypsinized and placed into a DQ‐gelatin/matrigel solution (25 µg/ml) for 24 h as previously outlined (Small *et al*, 2013). Cells were then fixed and stained for DAPI (blue), where indicated, before analysis by confocal microscopy.

### Plasma membrane repair analysis

Cell damage was evaluated using propidium iodide (PI) staining, as done before (Encarnação *et al*, 2016). Cells were washed with HBSS, trypsinized, and 200 ng/ml streptolysin O (SLO) (Sigma‐Aldrich, Steinheim, Germany) was added in HBSS (Ca^2+^ and Mg^2+^ free) containing DTT (10 mM). The SLO‐containing HBSS was removed and replaced by fresh HBSS (Ca^2+^ free) or EBSS (Ca^2+^ complete) for 10 min at 37°C. Cells were then stained using 50 μg/ml propidium iodide (PI) with RNase A (250 μg/ml), incubated at 37°C for 30 min, and analyzed by FACS Calibur (BD Biosciences, Franklin Lakes, USA) and FlowJo software.

### Cell lysis, pull downs, and immunoblotting

Cells were lysed in the following buffer: 25 mM Tris‐HCl pH 7.6, 150 mM NaCl, 1% NP‐40, 1% sodium deoxycholate, 0.1% SDS, supplemented with phenylmethylsulfonyl fluoride (1 mM), aprotinin (1.7 μg/ml), and leupeptin (10 μg/ml). Lysates were left on ice for 20 min, centrifuged at 15,000 *g* for 10 min at 4°C. Protein concentrations were determined and whole cell lysate was diluted into equal volumes and added to Laemlli buffer to a final concentration of 1× with 5% β‐mercaptoethanol (Sigma‐Aldrich, Steinheim, Germany). Where applicable, equal amounts of lysates were used to pull down HA‐tagged proteins using anti‐HA agarose before elution and the addition of Laemlli buffer. Subsequently, pull downs and lysate samples were boiled for 5 min at 95°C for protein denaturation. The samples were analyzed by SDS‐PAGE and Western blotting on PVDF membrane (Millipore, Waterford, UK). The membranes were then blocked in appropriate blocking agent, either 5% marvel or 3% BSA, in 0.1% Tween‐20/PBS for 1 h. After blocking, the membranes were probed with the indicated antibodies for 1 h at RT or overnight at 4°C. The following primary antibodies were used: anti‐HA (1:1,000, Thermo Fisher Scientific, Waltham, USA; Cat no: 715500), anti‐FLAG (1:1,000, Sigma‐Aldrich, Steinheim, Germany; Cat no: F3165), anti‐GAPDH (1:1000, Bio‐techne, Minneapolis, USA; Cat no: AF5718), anti‐USP17 (1:2,000, Fusion Antibodies, Belfast, UK; previously used in Burrows *et al*, 2009; de la Vega *et al*, 2011; Jaworski *et al*, 2014), anti‐cathepsin D (1:1,000, Abcam, Cambridge, UK; Cat no: ab75852), antitubulin (1:1,000, Abcam, Cambridge, UK; Cat no: ab6160), and anti‐LAMP1 (1:1,000, Abcam, Cambridge, UK; Cat no: ab25630). The membrane was incubated with the appropriate secondary antibody: either goat anti‐mouse HRP conjugate, or goat anti‐rabbit HRP conjugate (both diluted 1:10,000, Cell Signaling Technology, London, UK; Cat no: anti‐mouse (7076S), anti‐rabbit (7074S) ) or rabbit anti‐rat HRP conjugate (1:40,000, Abcam, Cambridge, UK; Cat no: ab205718). Proteins were detected with a chemiluminescence protocol and were exposed using the ChemiDoc XRS^+^ imaging system (BioRad, Hertfordshire, UK).

### RNA extraction and reverse transcription PCR

RNA was extracted using STAT‐60 according to the manufacturer's instructions (Tel‐Test Inc., Friendswood, USA). cDNAs were synthesized with the aid of a Transcriptor First Strand cDNA Synthesis kit (Roche Diagnostics, Indianapolis, USA). The Roche LightCycler 96 System was used for real‐time PCR analysis employing the cycle threshold (2−ΔΔCT) method. The following primers were used: USP17, 5′‐GGCTGCTGGCTTCCATAG‐3′ (forward) and 5′‐CCACAGTGAACATGAGAAATTCA‐3′ (reverse); RNF26, 5′‐TCGGCACTCAGAACCTCTTT‐3′ (forward) and 5′‐GCATCACCGACGGAAGGATC‐3′ (reverse); and GAPDH, 5′‐ATGGCAAATTCCATGGCA‐3′ (forward) and GAPDH 5′‐TCTAGACGGCAGGTCAGG‐3′ (reverse).

### Cathepsin D/E activity assay

HeLa cells were transfected as outlined and lysed using a non‐denaturing 50 mM sodium acetate lysis buffer (pH 5.5, 0.5 M EDTA, 0.2% Triton X‐100). Lysates were kept on ice for 30 min (short vortex every 10 min), then centrifuged at 15,000 *g* for 10 min at 4°C. Protein concentration was determined using BCA protein quantification assay. Five microgram of lysis protein in 50 µl of sodium acetate buffer was loaded onto a black bottomed 96‐well plate, and 50 µl of 20 µM Cathepsin D/E substrate (Mca‐Gly‐Lys‐Pro‐Ile‐Leu‐Phe‐Phe‐Arg‐Leu‐Lys(Dnp)‐D‐Arg‐NH2) (Enzo Life Sciences, Exeter, UK: BML‐P127) was added to each lysate sample. Samples with substrate were subsequently incubated at 37°C for 60 min, with fluorescence measured every 2 min (exc: 340 nm; em: 420 nm).

### Statistical analysis

Student’s *t*‐tests and one‐way ANOVA were calculated using the GraphPad software (Prism5). *P*‐values were considered statistically significant: *< 0.05, **< 0.01, ***< 0.001, and ****<0.0001. *P*‐values of > 0.05 were considered non‐significant (ns).

## Author contributions

**Jia Lin:** Data curation; Formal analysis. **Aidan P McCann:** Data curation; Formal analysis. **Naphannop Sereesongsaeng:** Data curation; Formal analysis. **Jonathan M Burden:** Data curation; Methodology. **Alhareth A Alsa'd:** Data curation; Methodology. **Roberta E Burden:** Conceptualization; Methodology. **Ileana Micu:** Conceptualization; Methodology. **Richard Williams:** Conceptualization; Methodology. **Sandra Van Schaeybroeck:** Conceptualization. **Emma Evergren:** Conceptualization. **Paul B Mullan:** Conceptualization. **Jeremy C Simpson:** Conceptualization. **Christopher J Scott:** Conceptualization. **James Frederick Burrows:** Conceptualization; Data curation; Supervision.

In addition to the CRediT author contributions listed above, the contributions in detail are:

JL, APM, NS, JMB, and AAA carried out the experimental work and participated in manuscript writing. IM assisted with the analysis of the microscopy data. REB, SVS, EE, RW, PM, JCS, and CJS participated in study design and manuscript writing. JFB conceived the study and participated in study design, coordination, and manuscript writing. All authors have read and approved the final manuscript.

## Supporting information



Expanded View Figures PDF

## Data Availability

Data sharing is not applicable to this article as no datasets were generated or analyzed during the current study.
