# Supplementary material for: USP17 is required for peripheral trafficking of lysosomes
Source: EMBO Rep. 2022 Jan 26;23(4):e51932. doi: 10.15252/embr.202051932 (PMC8982589; doi:10.15252/embr.202051932)
Supplement: Supplementary file 1 — Expanded View Figures PDF [file EMBR-23-e51932-s001.pdf]

Expanded View Figures

Figure EV1. USP17 shRNAs and RNF26 siRNAs deplete USP17 and RNF26 mRNA levels efficiently.

A HeLa and MDA-MB-231 cells were transfected with constructs for non-targeting shRNA, USP17 shRNA1, or USP17 shRNA2. QPCR was carried out to determine relative abundance of USP17 mRNA. The results plotted are representative of results obtained in three separate experiments.

B HeLa and MDA-MB-231 cells were transfected as in A. After 72 h, transfected cells were treated with Bortezomib (100 nM) for 6 h. Cell lysates were prepared and equal amounts of lysate were immunoprecipitated using anti-USP17 antibody. Immunoprecipitations were immunoblotted with anti-USP17 antibody, to confirm USP17 depletion.

C HeLa cells were transfected as in A, in conjunction with non-targeting control siRNA and RNF26 siRNA as indicated. QPCR was carried out to determine relative abundance of RNF26 mRNA. The results plotted are representative of results obtained in three separate experiments.

Data information: Total RNA was extracted for cDNA synthesis and real-time PCR analysis used the cycle threshold ($2-\Delta\Delta CT$) method ($n = 3$). Error bars represent standard errors.

© 2022 The Authors

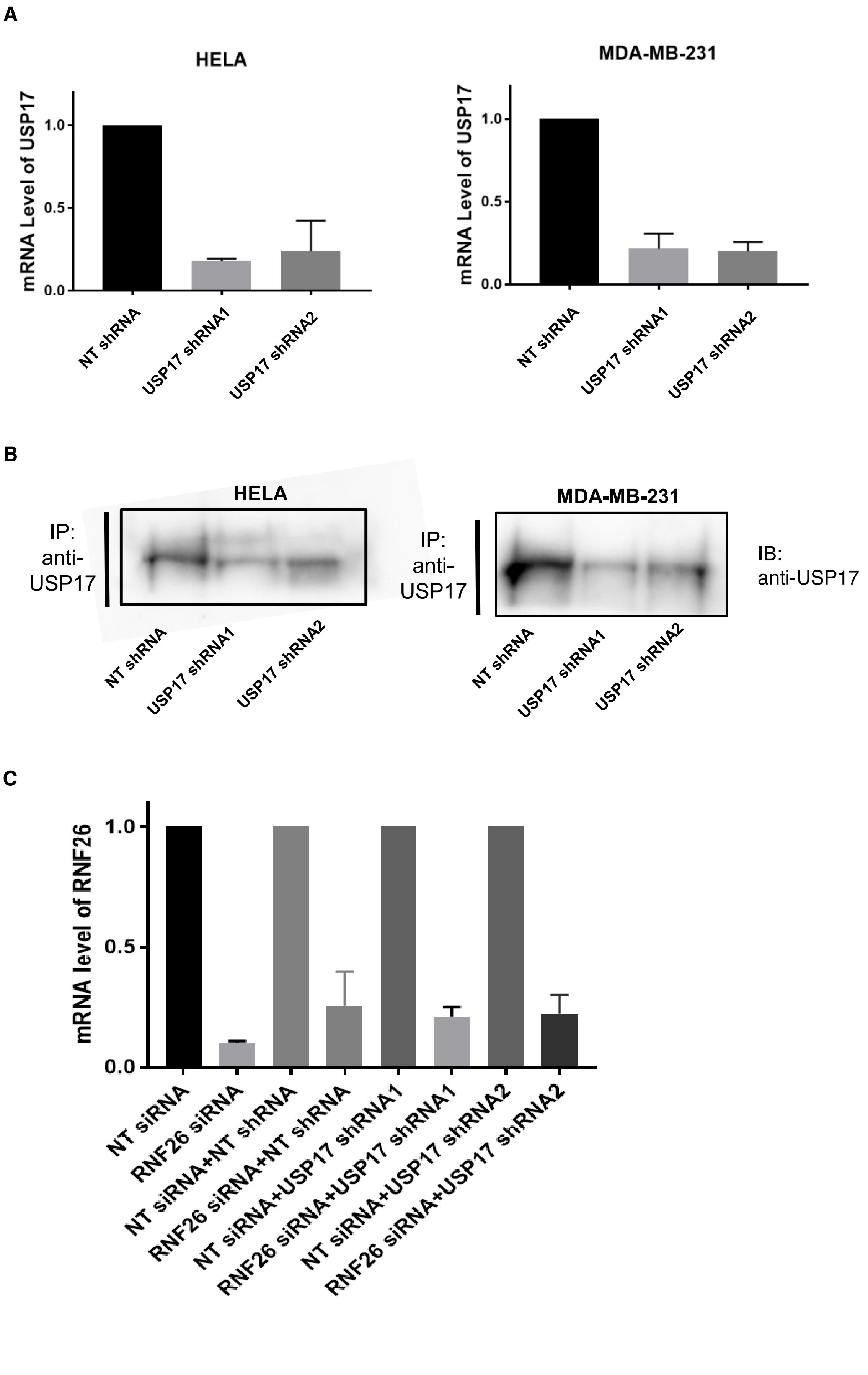

Figure EV1.

 © 2022 The Authors

Figure EV2. USP17 is necessary for peripheral lysosome trafficking.

A MDA-MB-231 cells were transfected with constructs for non-targeting (NT) shRNA, USP17 shRNA1, or USP17 shRNA2. Seventy-two hours post-transfection, the cells were stained for LAMP1 and the nuclei counterstained with DAPI.

B HeLa cells were transfected with empty vector, or expression constructs for USP17, or USP17CS (inactive mutant). Forty-eight hours post-transfection, lysates were harvested and immunoblotted for USP17, and LAMP1, as indicated.

C HeLa cells were transfected as in A in conjunction with a construct for GFP-tagged LAMP1. Seventy-two hours post-transfection, the nuclei were counterstained with DAPI.

Data information: In A and C, lower panels are enlarged images of the indicated area in the top panels and the cell membrane is marked by dotted line. Scale bars 25 µm.

© 2022 The Authors

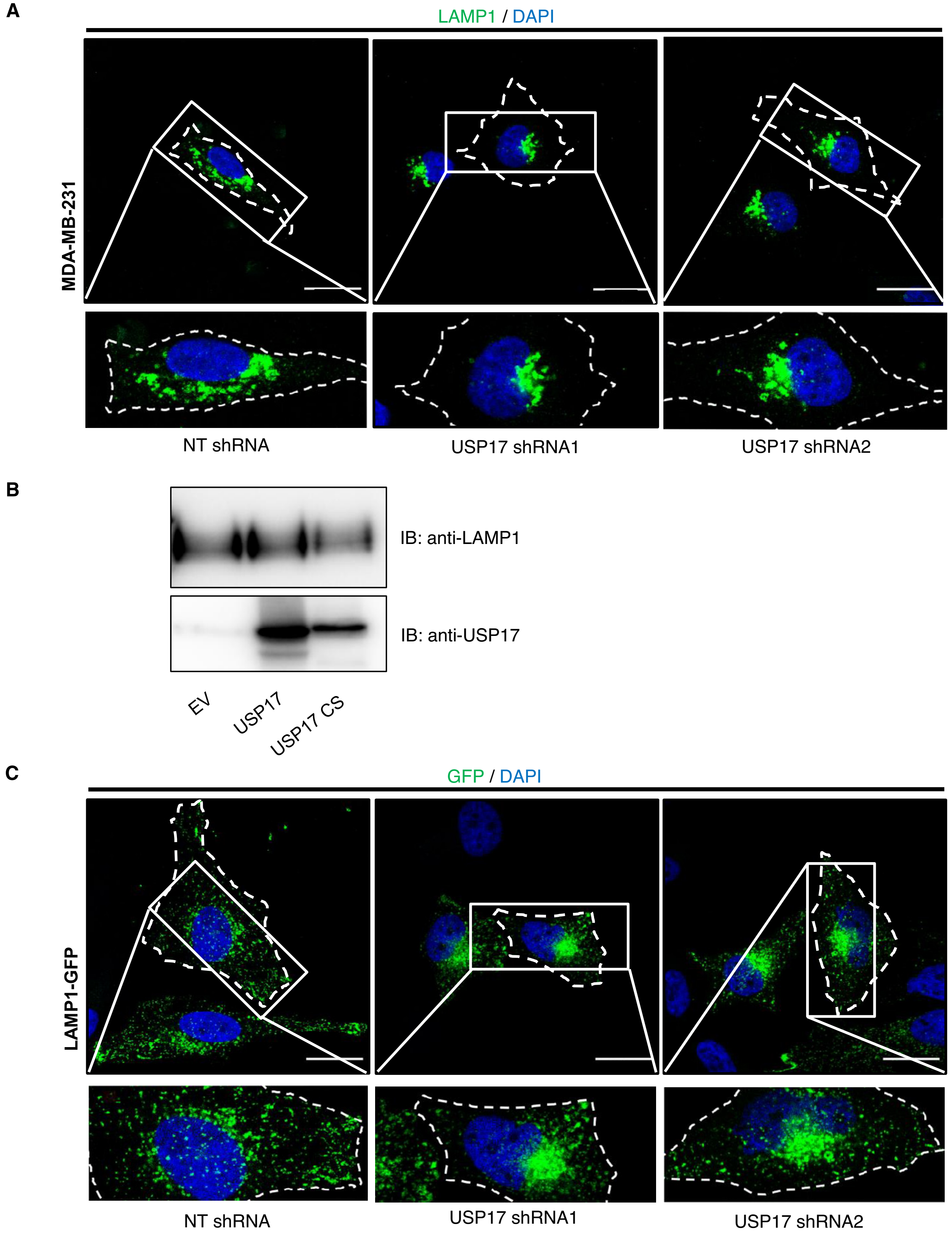

Figure EV2.

 © 2022 The Authors

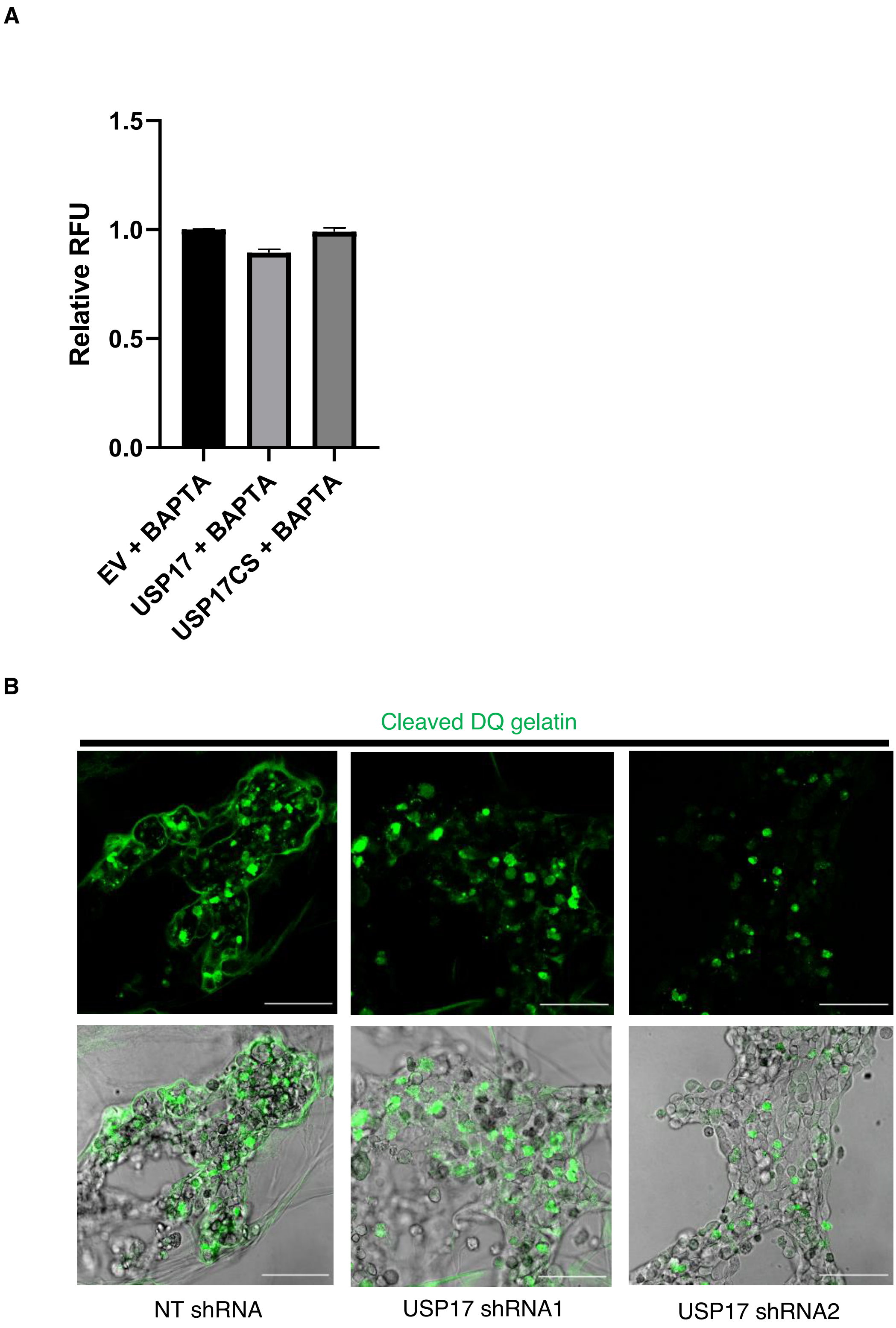
A
Relative RFU
1.5
1.0
0.5
0.0
EV + BAPTA
USP17 + BAPTA
USP17CS + BAPTA
B
Cleaved DQ gelatin
NT shRNA
USP17 shRNA1
USP17 shRNA2

Figure EV3.

Figure EV3. USP17 expression triggers secretion of lysosomal proteases.

A HeLa cells were transfected with empty vector, or expression constructs for USP17, or USP17CS (inactive mutant), as indicated. Cells were treated for 3 h with BAPTA-AM (30 μM) prior to making lysates. Forty-eight hours post-transfection, lysates were harvested and 5 μg of protein used in a CatD/E activity assay. RFU of triplicate samples at 60 min compared to empty vector control are plotted. The results plotted are representative of results obtained in three separate experiments. Error bars represent standard error.

B HeLa cells were transfected with constructs for non-targeting (NT) shRNA, USP17 shRNA1, or USP17 shRNA2. Seventy-two hours post-transfection, the cells were incubated in a DQ-gelatin/matrigel solution (25 μg/ml) to compare the proteolytic ability of the transfected cells. Proteolysis was then assessed in fluorescent (top panel) and bright-field and fluorescent images (bottom panel) taken using confocal microscopy. Scale bars 100 μm.

Figure EV4. USP17 is necessary for EGF-mediated peripheral lysosome trafficking.

A HeLa cells were transfected with constructs coding a non-targeting (NT) shRNA, USP17 shRNA1, or USP17 shRNA2 in conjunction with GFP-tagged CD63 as indicated. Forty-eight hours post-transfection, the cells were either serum starved (upper panels), or placed in serum-free medium with 100 μg/ml EGF (lower panels) for 16 h prior to being stained with DAPI. Right hand panels are enlarged images of the indicated area in left panels and the cell membrane is marked by dotted line.

B The distribution of at least 400 GFP-positive vesicles from a number of cells (n) from a series of confocal images across three separate experiments was plotted as vesicle relative position (mean value is red bar). Error bars represent standard error and **** indicates P-values < 0.0001. One-way ANOVA was used to determine statistically significant differences between groups.

 © 2022 The Authors

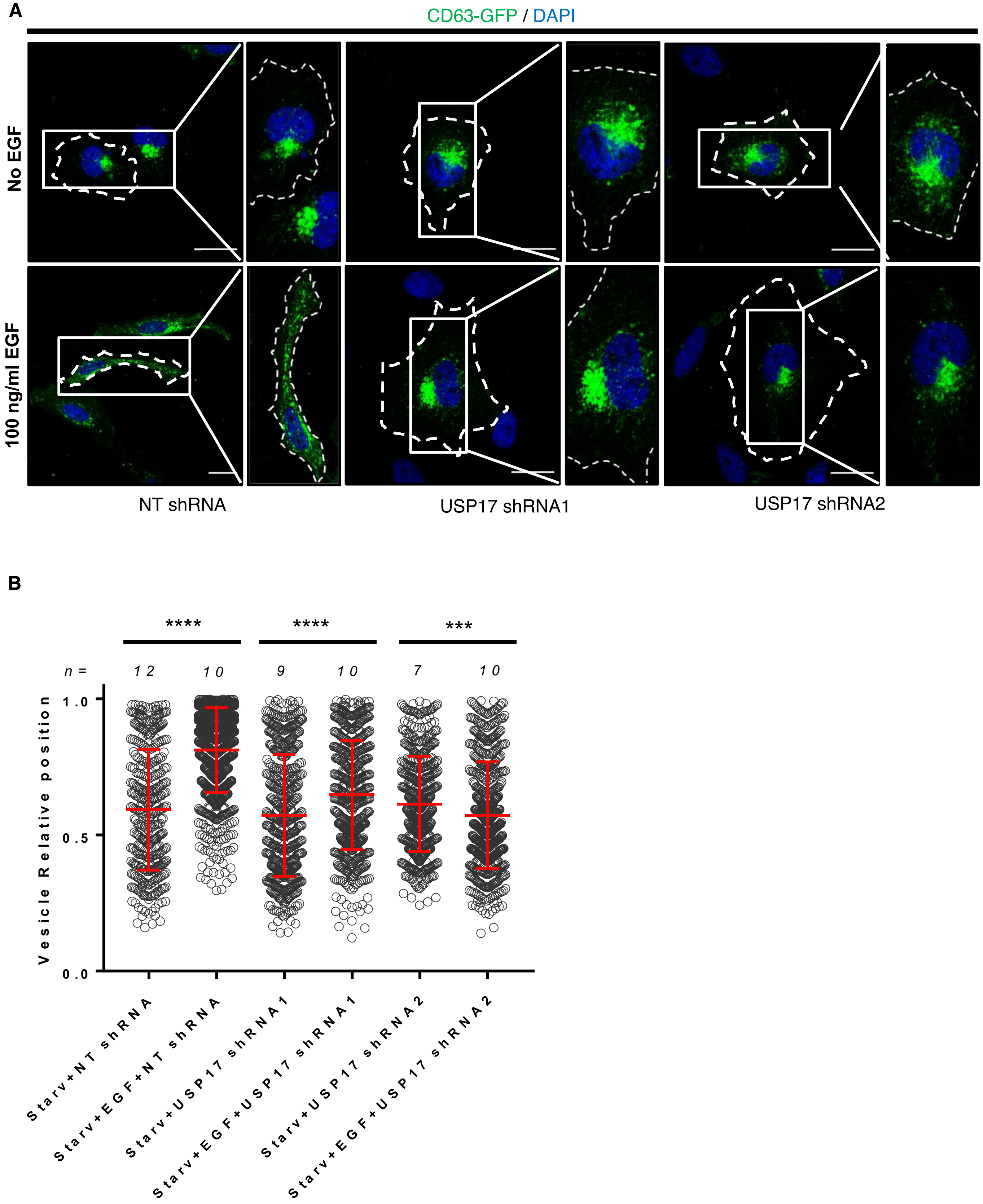
A
CD63-GFP / DAPI
No EGF
100 ng/ml EGF
NT shRNA
USP17 shRNA1
USP17 shRNA2
B
****
****
***
n =
12
10
9
10
7
10
Vesicle Relative position
1.0
0.5
0.0
Starv+NT shRNA
Starv+EGF+NT shRNA
Starv+USP17 shRNA1
Starv+EGF+USP17 shRNA1
Starv+USP17 shRNA2
Starv+EGF+USP17 shRNA2

Figure EV4.

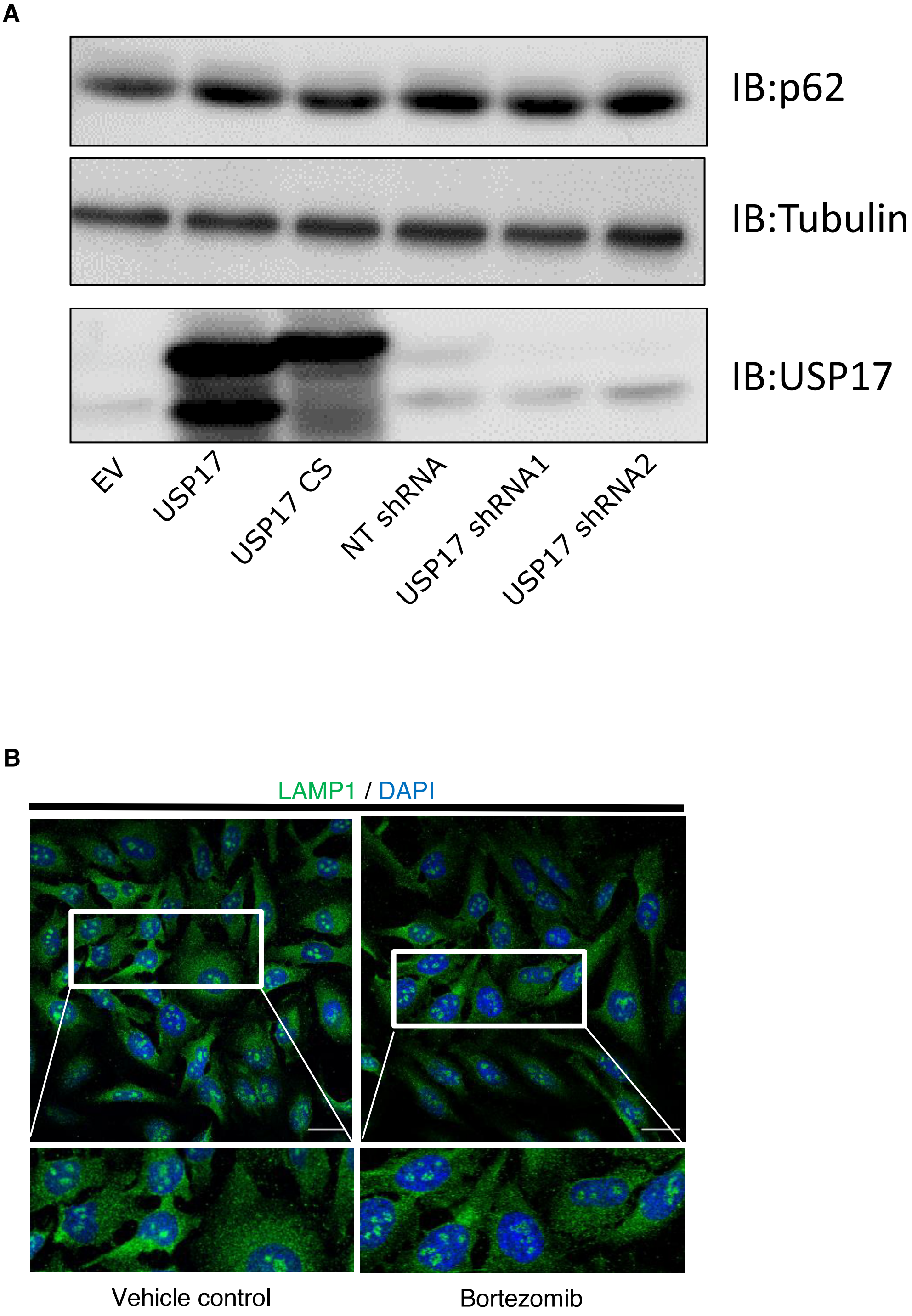

Figure EV5. USP17 does not impact upon p62 levels via the proteasome.

A HeLa cells were transfected with empty vector, or expression constructs for USP17 and USP17CS (inactive mutant), or coding a non-targeting (NT) shRNA, USP17 shRNA1, or USP17 shRNA2, as indicated. Forty-eight hours post-transfection, lysates were harvested and immunoblotted for tubulin, USP17, and p62, as indicated.

B HeLa cells were treated with vehicle control, or Bortezomib (100 nM), as indicated. After 6 h, cells were stained for LAMP-1 (green) and DAPI (blue). Lower panels are enlarged images of indicated area in top panels. Scale bars 25 μm.

 © 2022 The Authors
