# Supplementary material for: USP17 is required for peripheral trafficking of lysosomes
Source: EMBO Rep. 2022 Jan 26;23(4):e51932. doi: 10.15252/embr.202051932 (PMC8982589; doi:10.15252/embr.202051932)
Supplement: Supplementary file 2 [file EMBR-23-e51932-s002.pdf]

EMBO reports

USP17 is required for peripheral trafficking of lysosomes

Jia Lin, Aidan McCann, Naphannop Sereesongsaeng, Jonathan Burden, Alhareth Alsa'd, Roberta Burden, Ileana Micu, Richard Williams, Sandra Van Schaeybroeck, Emma Evergren, Paul Mullan, Jeremy Simpson, Christopher Scott, and James Burrows
DOI: 10.15252/embr.202051932

Corresponding author(s): James Burrows (j.burrows@qub.ac.uk)

Review Timeline:

Submission Date:	20th Oct 20
Editorial Decision:	27th Nov 20
Revision Received:	6th Oct 21
Editorial Decision:	26th Nov 21
Revision Received:	14th Dec 21
Accepted:	23rd Dec 21

Editor: Martina Rembold

Transaction Report:

(Note: With the exception of the correction of typographical or spelling errors that could be a source of ambiguity, letters and reports are not edited. Depending on transfer agreements, referee reports obtained elsewhere may or may not be included in this compilation. Referee reports are anonymous unless the Referee chooses to sign their reports.)

1st Editorial Decision 27th Nov 2020

Dear Dr. Van Schaeybroeck

Thank you for the submission of your research manuscript to our journal. We have now received the full set of referee reports that is copied below.

As you will see, the referees acknowledge that the findings are potentially interesting, but they also point out several technical concerns and have a number of suggestions for how the study should be strengthened, and I think that all of them should be addressed.

Given these constructive comments, we would like to invite you to revise your manuscript with the understanding that the referee concerns (as detailed above and in their reports) must be fully addressed and their suggestions taken on board. Please address all referee concerns in a complete point-by-point response. Acceptance of the manuscript will depend on a positive outcome of a second round of review. It is EMBO reports policy to allow a single round of revision only and acceptance or rejection of the manuscript will therefore depend on the completeness of your responses included in the next, final version of the manuscript.

We invite you to submit your manuscript within three months of a request for revision. This would be February 27th in your case. However, we are aware of the fact that many laboratories are not fully functional due to COVID-19 related shutdowns and we have therefore extended the revision time for all research manuscripts under our scooping protection to allow for the extra time required to address essential experimental issues. Please contact us if you want to discuss the time needed and the revisions further.

***** IMPORTANT NOTE: we perform an initial quality control of all revised manuscripts before re-review. Your manuscript will FAIL this control and the handling will be DELAYED if the following APPLIES:

1) A data availability section is missing.
2) Your manuscript contains error bars based on n=2. Please use scatter blots showing the individual datapoints in these cases. The use of statistical tests needs to be justified.

When submitting your revised manuscript, please carefully review the instructions that follow below. Failure to include requested items will delay the evaluation of your revision. ******

When submitting your revised manuscript, we will require:

1) a .docx formatted version of the manuscript text (including legends for main figures, EV figures and tables). Please make sure that the changes are highlighted to be clearly visible.

2) individual production quality figure files as .eps, .tif, .jpg (one file per figure).
Please download our Figure Preparation Guidelines (figure preparation pdf) from our Author Guidelines pages https://www.embopress.org/page/journal/14693178/authorguide for more info on how to prepare your figures.

3) a .docx formatted letter INCLUDING the reviewers' reports and your detailed point-by-point responses to their comments. As part of the EMBO Press transparent editorial process, the point-by-point response is part of the Review Process File (RPF), which will be published alongside your paper.

4) a complete author checklist, which you can download from our author guidelines (). Please insert information in the checklist that is also reflected in the manuscript. The completed author checklist will also be part of the RPF.

5) Please note that all corresponding authors are required to supply an ORCID ID for their name upon submission of a revised manuscript (). Please find instructions on how to link your ORCID ID to your account in our manuscript tracking system in our Author guidelines
()

6) We replaced Supplementary Information with Expanded View (EV) Figures and Tables that are collapsible/expandable online. A maximum of 5 EV Figures can be typeset. EV Figures should be cited as 'Figure EV1, Figure EV2" etc... in the text and their respective legends should be included in the main text after the legends of regular figures.

- For the figures that you do NOT wish to display as Expanded View figures, they should be bundled together with their legends in a single PDF file called *Appendix*, which should start with a short Table of Content. Appendix figures should be referred to in the main text as: "Appendix Figure S1, Appendix Figure S2" etc. See detailed instructions regarding expanded view here:

- Additional Tables/Datasets should be labeled and referred to as Table EV1, Dataset EV1, etc. Legends have to be provided in a separate tab in case of .xls files. Alternatively, the legend can be supplied as a separate text file (README) and zipped together with the Table/Dataset file.

7) Please note that a Data Availability section at the end of Materials and Methods is now mandatory. In case you have no data that requires deposition in a public database, please state so instead of refereeing to the database.
See also < https://www.embopress.org/page/journal/14693178/authorguide#dataavailability>). Please note that the Data Availability Section is restricted to new primary data that are part of this study.

8) We would also encourage you to include the source data for figure panels that show essential data. Numerical data should be provided as individual .xls or .csv files (including a tab describing the data). For blots or microscopy, uncropped images should be submitted (using a zip archive if multiple images need to be supplied for one panel). Additional information on source data and instruction on how to label the files are available .

9) Our journal encourages inclusion of *data citations in the reference list* to directly cite datasets that were re-used and obtained from public databases. Data citations in the article text are distinct from normal bibliographical citations and should directly link to the database records from which the data can be accessed. In the main text, data citations are formatted as follows: "Data ref: Smith et al, 2001" or "Data ref: NCBI Sequence Read Archive PRJNA342805, 2017". In the Reference list, data citations must be labeled with "[DATASET]". A data reference must provide the database name, accession number/identifiers and a resolvable link to the landing page from which the data can be accessed at the end of the reference. Further instructions are available at .

10) Regarding data quantification
The following points must be specified in each figure legend:
- the name of the statistical test used to generate error bars and P values,
- the number (n) of independent experiments (please specify technical or biological replicates) underlying each data point,
- the nature of the bars and error bars (s.d., s.e.m.)
Discussion of statistical methodology can be reported in the materials and methods section, but figure legends should contain a basic description of n, P and the test applied.
- Please also include scale bars in all microscopy images.

11) As part of the EMBO publication's Transparent Editorial Process, EMBO reports publishes online a Review Process File to accompany accepted manuscripts. This File will be published in conjunction with your paper and will include the referee reports, your point-by-point response and all pertinent correspondence relating to the manuscript.

You are able to opt out of this by letting the editorial office know (emboreports@embo.org). If you do opt out, the Review Process File link will point to the following statement: "No Review Process File is available with this article, as the authors have chosen not to make the review process public in this case."

We would also welcome the submission of cover suggestions, or motifs to be used by our Graphics Illustrator in designing a cover.

I look forward to seeing a revised version of your manuscript when it is ready. Please let me know if you have questions or comments regarding the revision.

Yours sincerely

Martina Rembold, PhD
Senior Editor
EMBO reports

****

Referee #1:

The presented study by Burrows and colleagues investigated the impact of the deubiquitinase USP17 on lysosome positioning and trafficking. The authors showed that siRNA-mediated knockdown (KD) of USP17 prevented peripheral lysosome positioning. Overexpression of USP17 resulted in reduced intracellular cathepsin D levels and increased secretion of lysosomal proteases by lysosome exocytosis. This observation was dependent on USP17 catalytic activity. EGF-driven late endosome and lysosome peripheral trafficking was inhibited in USP17 KD cells as well. Furthermore, the authors established that USP17 is required for plasma membrane repair. Upon KD of USP17, cells were not capable to fully repair streptolysin-O-induced pores in the plasma membrane as shown by propidium iodide uptake assays. Mechanistically, the authors suggested that USP17 counteracts

RNF26-meditade ubiquitination of p62, a modification that allows p62 to act as a bridge between the ER and lysosomes and that tethers them in the perinuclear cloud.

Taken together, the study by Lin et al. presents a very interesting and topical finding with a broad biological significance; it establishes USP17 as important regulator of peripheral trafficking and exocytosis of lysosomes, which is necessary for EGF-triggered invasion and motility, and plasma membrane repair. Length and format of the manuscript are appropriate, and the drawn conclusions based on presented data are justified.

I recommend publication after addressing the following points:

- Complementary to the qPCR analysis in EV Fig. 1, the authors should verify the knockdown efficiency of both siRNAs against USP17 by Western blot. It would be good to know how much USP17 protein is still expressed. Alternatively, IF staining of USP17 could be included in Fig. 1A.

- What is known about the cellular localization of USP17? Does it localize to the perinuclear region, i.e. does it co-localize with its suggested substrate p62 at the lysosome? The authors should try to co-stain USP17 and LAMP1, RNF26 or p62.

Referee #2:

Evaluation of EMBOR-2020-51932V1

In their manuscript "USP17 is required for peripheral trafficking of lysosomes" by Jia Lin, Aidan McCann, Naphannop Sereesongsaeng, Roberta Burden, Ileana Micu, Richard Williams, Sandra Van Schaeybroeck, Emma Evergren, Paul Mullan, Jeremy Simpson, Christopher Scott, and James Burrows authors present evidence of the deubiquitinase USP17:s role as a regulator of peripheral trafficking of lysosomes. Authors show how depletion of USP17 with 2 specific shRNAs inhibit EGF-induced peripheral positioning of lysosomes and suggest that this is due to cells inability to deubiquitinate p62. The study is interesting, and the manuscript is well written but is requiring some clarifications.

Criticism:

1. The study gives implications for an interesting possibility, but it does not really show that the inhibition of deubiquitination of p62 is responsible for the changes in the positioning of lysosomes. The lysosome positioning studies are conducted by depletion of USP17 and the ability of USP17 to deubiquitinate p62 is done in overexpression studies where both USP17 and p62 are overexpressed. Thus, it would be good if authors can show that p62 associates with endogenous USP17.
2. It has been shown in HeLa cells that proteosome inhibition with BTZ leads into ubiquitination of p62. Can authors show that BTZ treatment leads to ubiquitination of p62 and affects lysosome positioning? What about EGF treatment? Does EGF treatment change the ubiquitination of p62?
3. What is the relation of autophagy with USP17? Is it possible that depletion of USP17 induces autophagy and this way results in redistribution of lysosomes? Is autophagy induced by depletion of USP17? EGF activation could be inducing mTOR and then depletion of USP17 could inhibit mTOR?
4. Authors use LAMP1 and CD63 staining as markers of lysosomes. Are the protein levels of LAMP1 and CD63 affected by the depletion of USP17?

Referee #3:

This is an interesting and well-performed study, and the overall quality of the data is high. I just have a few comments for the author's attention:

1) Fig. 1 F - Can these results be confirmed/expanded using one of the available commercial kits for cathepsin D activity? This would be useful because the western blots seem to suggest that more pro-cathepsin D than mature cathepsin D is secreted. The authors are correct to conclude that active enzyme is secreted, but a biochemical assay would allow a precise determination of what percentage of the total cathepsin D activity present in the cells (this value can be obtained from cell lysates) is released into the supernatant. In prior studies using other methods to trigger lysosomal exocytosis this number is usually around 10%, at the most. It would be interesting to see if USP17 overexpression triggers exocytosis of a larger fraction of the mature lysosome population.

2) Fig. 1G - This is a very interesting result, but can the results be quantified using image analysis in a larger number of cells? Alternatively, if biochemical assays confirm the enzyme secretion (point 1 above), showing a lower magnification image with more cells in the field would be sufficient.

3) Page 8, second paragraph - a primary paper (not a review) should be cited to support the statement: This allows secreted lysosomal proteases and acid sphingomyelinase to combine to produce ceramide at the plasma membrane, triggering

endocytosis of damaged membrane and restoring plasma membrane integrity.

4) Fig. 3A - The effect of USP17 depletion on plasma membrane repair is impressive. Which begs the question - is the effect of USP17 overexpression on lysosome enzyme secretion calcium-dependent? And does USP17 overexpression enhance plasma membrane repair? The SLO conditions used in Fig. 3A suggest that the experiment was done under conditions where most of the SLO lesions are repaired. But using increasingly higher doses of SLO it is possible to start to observe partial repair (see for example Tam C et al J. Cell Biol 189:1027-38, 2010), allowing the assessment of a potential enhancement by USP17.

5) The authors conclude that USP17 is able to counteract the ubiquitination of p62 by
RNF26, and untether LEs/lysosomes from the ER to allow their peripheral trafficking. So it should be possible to impact lysosome enzyme secretion and plasma membrane repair by manipulating these additional molecules - was this done?

Minor points:

1. The authors should indicate their source of SLO. Do they have access to the cysteine mutant that eliminates the need for reducting agents to trigger pore-forming activity?

2. Apparent typo on page 9: However, SLO treatment more than trebled the number of cells

QUEEN'S UNIVERSITY BELFAST | SCHOOL OF PHARMACY

Dr James Burrows
Lecturer in Pharmacology
School of Pharmacy
Queen's University Belfast
97 Lisburn Road
Belfast
Tel: +44 28 90972703
Fax: +44 28 90247794
j.burrows@qub.ac.uk

6th October, 2021

Dr Rembold,

Thank you for considering our paper entitled "USP17 is required for peripheral trafficking of lysosomes" for publication in EMBO reports. We are delighted that you and the reviewers think it is of interest and that you have invited us to submit a revised manuscript. We have now addressed the points outlined by the reviewers within the revised manuscript and in our responses below. Hopefully this will all be to everyone's satisfaction and we can look forward to the publication of this paper in EMBO reports.

Yours sincerely,

James Burrows

James Burrows

All alterations to the text have been indicated in red.

Referee #1:

1] The referee asked that complementary to the qPCR analysis in EV Fig. 1, the authors should verify the knockdown efficiency of both shRNAs against USP17 by Western blot.

A: In the revised manuscript we have now included Western blots illustrating the knockdown of USP17 protein in HeLa and MDA-MB-231 cells in EV Figure 1B, and mentioned these in the text on page 5, paragraph 1, line 3.

2] The referee asked what is known about the cellular localization of USP17? Does it localize to the perinuclear region, i.e. does it co-localize with its suggested substrate p62 at the lysosome?

A: We have previously demonstrated that endogenous USP17 shows a peri-nuclear distribution in HeLa cells (McFarlane et al., Cancer Res 2010) and shown that it co-localises with the endoplasmic reticulum (ER) marker calnexin (Burrows et al., JBC 2009), indicating it is associated with the ER. We

have emphasised this in the revised manuscript to clarify this point (Page 4, paragraph 1 of Results and Discussion, lines 3-5) and we have added an additional reference (Burrows et al., 2009) to the bibliography. RNF26 has also been localised to the ER, and shown to interact with, and co-localise with, p62 at this location (Jongsma et al., Cell 2016), indicating USP17, RNF26, and p62 are in close proximity.

Referee #2:

1] The referee asked if p62 associates with endogenous USP17.

A: As mentioned in the answer to referee 1, we have now emphasised that we have previously demonstrated that endogenous USP17 shows a peri-nuclear distribution in HeLa cells (McFarlane et al., Cancer Res 2010) and shown that it co-localises with the endoplasmic reticulum (ER) marker calnexin (Burrows et al., JBC 2009), indicating it is in close proximity to RNF26, which has also been localised to the ER (Jongsma et al., Cell 2016). In addition, RNF26 has also been shown to bind to, and co-localise with p62, at the ER, which would also place this substrate in close proximity to USP17 (Jongsma et al., 2016). As suggested by the reviewer, we examined if USP17 directly interacted with p62 in immunoprecipitations. However, although we did not see evidence of a direct interaction, USP17 could easily deubiquitinate p62 tethered by RNF26 in close proximity on the ER.

2] The referee indicated in HeLa cells that proteosome inhibition with BTZ leads to ubiquitination of p62 and asked if BTZ treatment affects lysosome positioning?

A: The previous study examining RNF26 has shown it mono-ubiquitinates p62, and indicated the mono-ubiquitinated p62 acts as a bridge to tether the vesicles to the ER (Jongsma et al., 2016). This would indicate the mechanism is unlikely to involve targeting p62 to the proteasome, and this is supported by EV Figure 5A, which demonstrates that USP17 overexpression and depletion does not impact upon endogenous p62 levels. To further confirm this we examined the impact of Bortezomib (BTZ) on lysosome position and found that it does not have any marked impact upon LAMP1 localisation (EV Figure 5B). These points are now mentioned in the text (page 11, final paragraph, lines 7-12; page 12, lines 1-2)

3] The referee asks if USP17 impacts upon autophagy and if this could explain the redistribution of lysosomes?

A: As mentioned above, USP17 does not impact upon endogenous p62 levels (EV Figure 5A), a marker of autophagic flux. This indicates USP17 does not impact upon autophagy, and we have made reference to this in the text on page 11, final paragraph, lines 7-12.

4] The referee asks if LAMP1 and CD63 protein levels could be affected by the depletion of USP17?

A: In EV Figure 2B we have shown that USP17 has no marked impact upon the levels of LAMP1 protein, indicating this does not explain the impact upon lysosomal localisation. We have mentioned this in the text on page 5, paragraph 1, lines 14-17. This fits with previous observations in regard to RNF26, which also did not impact upon LAMP1 levels (Jongsma et al., 2016), and our new data showing BTZ has no impact upon LAMP1 localisation (EV Figure 5B).

Referee #3:

1] The referee asked if the results in Figure 1F could be confirmed/expanded by examining cathepsin D activity? The referee thinks a biochemical assay would allow a precise determination of what percentage of the total cathepsin D activity present in the cells is released into the supernatant.

A: We have undertaken an assay using a cathepsin D/E specific substrate, and lysates from cells overexpressing USP17, and this is now included in the new Figure 1G. Expression of USP17, but not its inactive mutant, resulted in a drop in intracellular cathepsin D/E activity (approximately 40%), which indicates the observed increase in extracellular protease activity observed in EV Figure 3B is due to secretion of lysosomal proteases. This indicates USP17 expression can facilitate secretion of lysosomal proteases, including cathepsin D/E. We have also discussed this result in the text on page 6, paragraph 2, lines 6-12.

2] The referee asked that we show a lower magnification image for Figure 1G with more cells present.

A: We have redone the experiments previously illustrated in the original Figure 1G, and included lower magnification images in EV Figure 3B of the revised manuscript, and made reference to this in the text (page 7, paragraph 1, line 7).

3] The referee asked on page 8, second paragraph, that we use a primary paper (not a review) to support the statement: This allows secreted lysosomal proteases and acid sphingomyelinase to combine to produce ceramide at the plasma membrane, triggering endocytosis of damaged membrane and restoring plasma membrane integrity.

A: We thank the reviewer for pointing out this oversight and we have now included a more in depth explanation of this process and cited original articles. In particular, we have mentioned that lysosomal exocytosis is required for PMR (Reddy et al., Cell 2001) and that secretion of the lysosomal enzyme acid sphingomyelinase is required to produce ceramide triggering endocytosis of damaged membrane (Tam et al., J. Cell Biol 2010). In addition, we have mentioned that this process is regulated by the secretion of lysosomal proteases including Cathepsins B, D and L (Castro-Gomes et al., PLoS One 2016). All of these references have also been added to the bibliography. (Page 8, paragraph 3, lines 6-10; page 9, line 1)

4] The referee asks if the effect of USP17 overexpression on lysosome enzyme secretion is calcium-dependent and if USP17 overexpression enhances plasma membrane repair?

A: We examined the impact of USP17 overexpression upon SLO mediated damage. However, when we titrated up the amount of SLO used, as suggested by the reviewer, we lost a large proportion of the cells, probably due to excessive damage compromising the cell membranes. As a result, it was difficult to interpret the results obtained, but we didn't see anything to suggest USP17 overexpression impacts upon the cells ability to repair SLO mediated damage. We also looked at the impact of calcium on USP17 driven secretion, by observing the impact of the intracellular calcium chelator BAPTA-AM upon cathepsin D/E activity. In the presence of BAPTA-AM, the impact of USP17 upon cathepsin D/E activity was markedly blunted, indicating the action of USP17 is calcium dependent (EV Figure 3A). This may indicate USP17 facilitates the transport of the lysosomes to the cell periphery, but still requires a calcium dependent process to allow their fusion with the plasma membrane.

5] The referee asks if manipulating p62 or RNF26 can impact upon lysosome enzyme secretion and plasma membrane repair?

A: p62 has many roles, such as in autophagy, in addition to its role in tethering vesicles, and so manipulating its levels is likely to impact all of these functions, making the results hard to interpret. RNF26 depletion has been shown in this paper (Figure 4A), and in previous work (Jongsma et al., 2016), to disperse lysosomes to the cell periphery, and more importantly, in the absence of RNF26, USP17 depletion has no impact upon lysosome positioning. We therefore believe that our results already clearly show the connection between USP17 and RNF26.

Minor points:

1] The referee asked that we indicate our source of SLO, and if we had access to the cysteine mutant that eliminates the need for reducing agents to trigger pore-forming activity?

A: We obtained the SLO from Sigma as a lyophilised powder, which we have now indicated in the materials and methods and followed the manufacturers instructions when dissolving the SLO for use. We did not have access to a cysteine mutant of SLO. However, we did fail to mention that we added DTT to the SLO containing medium prior to adding it to the cells to prompt damage, something which we have now corrected (page 17, final paragraph).

2] The referee pointed out a typo on page 9: However, SLO treatment more than trebled the number of cells.

A: This refers to Figure 3A where the introduction of SLO, in the absence of calcium, results in an elevation in the number of cells taking up PI, which indicates the presence of damage. In all three cases the introduction of SLO results in the percentage of cells demonstrating damage rising 3 fold, hence why this refers to SLO treatment trebling the number of cells damaged. However, to clarify further we have altered the text to indicate it altered the percentage of cells demonstrating damage (page 9, lines 16-19).

In addition to addressing the referees comments, we have also added two new references relating to RNF26, to further emphasise the link between USP17 and RNF26, both of which have been published since our original submission, and discussed these in the text (page 12, final line and page 13, paragraph 1). We have also added any additional methods required for the new experiments, altered the figure legends as required and corrected a few typographical errors we noticed ourselves.

1st Revision - Editorial Decision 26th Nov 2021

Dear Dr. Burrows

Thank you for the submission of your revised manuscript to EMBO reports. I apologize for the delay in handling your manuscript, but we have now received the two enclosed reports on it.

As you will see, both referees are very positive about the study and support publication.

Browsing through the manuscript myself, I noticed a few editorial things that we need before we can proceed with the official acceptance of your study.

- Please submit the EV figures as individual files.

- Please add the catalogue number or another identifier for the antibodies in the methods section.

- References: Please remove the bullet points and list only up to 10 authors (followed by et al).

- Please add information on funding to the manuscript (Acknowledgment section).

- We generally recommend arranging the figures so that the panels can be called out in an alphabetical order. In this respect we notice that Fig 1E is called out directly after 1A.

- Please remove the numbers from the scale bars in the images and only define their size in the legend.

- I attach to this email a related manuscript file with comments by our data editors. Please address all comments and upload a revised file with tracked changes with your final manuscript submission. Please note that statistical comparison should be limited to comparing data from biological replicates and not be applied to technical replicates.

- Finally, EMBO reports papers are accompanied online by A) a short (1-2 sentences) summary of the findings and their significance, B) 2-3 bullet points highlighting key results and C) a synopsis image that is 550x200-600 pixels large (width x height) in .png format. You can either show a model or key data in the synopsis image. Please note that the size is rather small and that text needs to be readable at the final size. Please send us this information along with the revised manuscript.

We look forward to seeing a final version of your manuscript as soon as possible. Please use this link to submit your revision: https://embor.msubmit.net/cgi-bin/main.plex

Kind regards

Martina Rembold, PhD
Senior Editor
EMBO reports

****

Referee #1:

The authors have addresed all comments and concerns raised by the reviewers. I recommend publication of the revised manuscript.

Referee #2:

The authors have sufficiently addressed the criticism.

The authors have addressed all minor editorial requests.

Dr. James Burrows
Queens University Belfast
School of Pharmacy
97 Lisburn Road
Belfast BT9 7BL
United Kingdom

Dear Dr. Burrows,

I am very pleased to accept your manuscript for publication in the next available issue of EMBO reports. Thank you for your contribution to our journal.

At the end of this email I include important information about how to proceed. Please ensure that you take the time to read the information and complete and return the necessary forms to allow us to publish your manuscript as quickly as possible.

As part of the EMBO publication's Transparent Editorial Process, EMBO reports publishes online a Review Process File to accompany accepted manuscripts. As you are aware, this File will be published in conjunction with your paper and will include the referee reports, your point-by-point response and all pertinent correspondence relating to the manuscript.

If you do NOT want this File to be published, please inform the editorial office within 2 days, if you have not done so already, otherwise the File will be published by default [contact: emboreports@embo.org]. If you do opt out, the Review Process File link will point to the following statement: "No Review Process File is available with this article, as the authors have chosen not to make the review process public in this case."

Should you be planning a Press Release on your article, please get in contact with emboreports@wiley.com as early as possible, in order to coordinate publication and release dates.

Thank you again for your contribution to EMBO reports and congratulations on a successful publication. Please consider us again in the future for your most exciting work.

Yours sincerely,

Martina Rembold, PhD
Senior Editor
EMBO reports

****

THINGS TO DO NOW:

You will receive proofs by e-mail approximately 2-3 weeks after all relevant files have been sent to our Production Office; you should return your corrections within 2 days of receiving the proofs.

Please inform us if there is likely to be any difficulty in reaching you at the above address at that time. Failure to meet our deadlines may result in a delay of publication, or publication without your corrections.

All further communications concerning your paper should quote reference number EMBOR-2020-51932V3 and be addressed to emboreports@wiley.com.

Should you be planning a Press Release on your article, please get in contact with emboreports@wiley.com as early as possible, in order to coordinate publication and release dates.

EMBO PRESS

YOU MUST COMPLETE ALL CELLS WITH A PINK BACKGROUND ↓

PLEASE NOTE THAT THIS CHECKLIST WILL BE PUBLISHED ALONGSIDE YOUR PAPER

Corresponding Author Name: James Burrows
Journal Submitted to: EMBO reports
Manuscript Number: EMBOR-2020-51932V2

Reporting Checklist For Life Sciences Articles (Rev. June 2017)

This checklist is used to ensure good reporting standards and to improve the reproducibility of published results. These guidelines are consistent with the Principles and Guidelines for Reporting Preclinical Research issued by the NIH in 2014. Please follow the journal's authorship guidelines in preparing your manuscript.

A- Figures
1. Data
The data shown in figures should satisfy the following conditions:

- the data were obtained and processed according to the field's best practice and are presented to reflect the results of the experiments in an accurate and unbiased manner.
- figure panels include only data points, measurements or observations that can be compared to each other in a scientifically meaningful way.
- graphs include clearly labeled error bars for independent experiments and sample sizes. Unless justified, error bars should not be shown for technical replicates.
- if n< 5, the individual data points from each experiment should be plotted and any statistical test employed should be justified
- Source Data should be included to report the data underlying graphs. Please follow the guidelines set out in the author ship guidelines on Data Presentation.

2. Captions
Each figure caption should contain the following information, for each panel where they are relevant:

- a specification of the experimental system investigated (eg cell line, species name).
- the assay(s) and method(s) used to carry out the reported observations and measurements
- an explicit mention of the biological and chemical entity(ies) that are being measured.
- an explicit mention of the biological and chemical entity(ies) that are altered/varied/perturbed in a controlled manner.

- the exact sample size (n) for each experimental group/condition, given as a number, not a range;
- a description of the sample collection allowing the reader to understand whether the samples represent technical or biological replicates (including how many animals, litters, cultures, etc.).
- a statement of how many times the experiment shown was independently replicated in the laboratory.
- definitions of statistical methods and measures:
  - common tests, such as t-test (please specify whether paired vs. unpaired), simple χ2 tests, Wilcoxon and Mann-Whitney tests, can be unambiguously identified by name only, but more complex techniques should be described in the methods section;
  - are tests one-sided or two-sided?
  - are there adjustments for multiple comparisons?
  - exact statistical test results, e.g., P values = x but not P values < x;
  - definition of 'center values' as median or average;
  - definition of error bars as s.d. or s.e.m.

Any descriptions too long for the figure legend should be included in the methods section and/or with the source data.

In the pink boxes below, please ensure that the answers to the following questions are reported in the manuscript itself. Every question should be answered. If the question is not relevant to your research, please write NA (non applicable). We encourage you to include a specific subsection in the methods section for statistics, reagents, animal models and human subjects.

USEFUL LINKS FOR COMPLETING THIS FORM

http://www.antibodypedia.com
http://1degreebio.org
http://www.equator-network.org/reporting-guidelines/improving-bioscience-research-repor
http://grants.nih.gov/grants/olaw/olaw.htm
http://www.mrc.ac.uk/Ourresearch/Ethicsresearchguidance/Useofanimals/index.htm
http://ClinicalTrials.gov
http://www.consort-statement.org
http://www.consort-statement.org/checklists/view/32-consort/66-title
http://www.equator-network.org/reporting-guidelines/reporting-recommendations-for-tumo
http://datadryad.org
http://figshare.com
http://www.ncbi.nlm.nih.gov/gap
http://www.ebi.ac.uk/ega
http://biomodels.net/
http://biomodels.net/miriam/
http://jjj.biochem.sun.ac.za
http://oba.od.nih.gov/biosecurity/biosecurity_documents.html
http://www.selectagents.gov/

B- Statistics and general methods

Please fill out these boxes ↓ (Do not worry if you cannot see all your text once you press return)

1.a. How was the sample size chosen to ensure adequate power to detect a pre-specified effect size?	For Figures 1B, 1E, 2B, 4B, 5B and EV4B we were examining the relative vesicle position, and comparing cell populations. To estimate how many vesicles/cells we required we used the Biomath site (http://biomath.info/power/) to estimate the population required to give us statistical Power of 80%. This gave us a minimum vesicle populations required of approximately 20, but in all cases we had annotated a population far in excess of what was required. For Figures EV1A/C we were examining the relative expression of mRNA using quantitative RT-PCR. It is standard practice to use three replicates in each experiment, and so sample size was not an issue. For Figures 1G, and EV3A we were examining the relative RFU in a cathepsin D/E activity assay. It is standard practice to use three replicates in each experiment, and so sample size was not an issue.
1.b. For animal studies, include a statement about sample size estimate even if no statistical methods were used.	NA
2. Describe inclusion/exclusion criteria if samples or animals were excluded from the analysis. Were the criteria pre-established?	NA
3. Were any steps taken to minimize the effects of subjective bias when allocating animals/samples to treatment (e.g. randomization procedure)? If yes, please describe.	NA
For animal studies, include a statement about randomization even if no randomization was used.	NA
4.a. Were any steps taken to minimize the effects of subjective bias during group allocation or/and when assessing results (e.g. blinding of the investigator)? If yes please describe.	NA
4.b. For animal studies, include a statement about blinding even if no blinding was done	NA
5. For every figure, are statistical tests justified as appropriate?	In Figures 1B, 1E, 2B, 4B, 5B and EV4B we used graphpad to undertake a one way ANOVA to compare multiple indepedent populations to determine if there was evidence of statistically significant differences. Then to assess which populations differed and how significant any differences were, we used a Tukey-Kramer test to determine which groups were significantly different. This test was justified as we were comparing three groups, or more, from unrelated populations.
Do the data meet the assumptions of the tests (e.g., normal distribution)? Describe any methods used to assess it.	The main assumptions made for the one way ANOVA, and so the Tukey-Kramer test, is that the populations are independent, they are normally distributed, and they have a similar variance. In Figures 1B, 1E, 2B, 4B, 5B and EV4B the various populations are independent, but when we used graphpad to examine the normality of the data, it indicated the majority of the populations were not normally distributed, and although there were differences in variance, graphpad indicated there were no outliers. The one-way ANOVA is robust for validity against nonnormality, but it may reduce the power of the test and this is a consideration when using it. However, this is a standard test for these types of experiments and similar methods were used in previous studies to determine differences in the relative vesicle position of different populations (Jongsma et al. [2016] Cell 166, 152-166).

Is there an estimate of variation within each group of data?	In Figures 1B, 1D, 2B, 4B, 5B and EV4B the error bars illustrate the standard error, which provides an estimate of the variation within in each group. In Figures 1G, EV1A/C, and EV3A, the error bars also illustrate the standard error, which provides an estimate of the variation within in each group.
Is the variance similar between the groups that are being statistically compared?	In Figures 1B, 1E, 2B, 4B, 5B and EV4B graphpad analysis indicated there were differences in variance between the populations, although it also indicated there were no outliers.

C- Reagents

6. To show that antibodies were profiled for use in the system under study (assay and species), provide a citation, catalog number and/or clone number, supplementary information or reference to an antibody validation profile. e.g., Antibodypedia (see link list at top right), 1DegreeBio (see link list at top right).	anti-HA polyclonal SG77 (Thermo fisher scientific, Cat no: 715500), anti-FLAG monoclonal M2 (Sigma-Aldrich, Cat no: F3165), anti-GAPDH (Bio-techne, Cat no: AF5718), anti-USP17 (Fusion Antibodies, Citations: Jaworski et al. (2014) Oncotarget, 5, 6964-6975; Jaworski et al. (2014) Biochem J, 457, 289-300; de la Vega et al. (2011) Nat Commun, 2, 259), anti-cathepsin D [EPR3057Y] (Abcam, Cat no: ab75852), anti-tubulin (Abcam, Cat no:ab6160), anti-LAMP1 [H4A3] (Abcam, Cat no: ab25630).
7. Identify the source of cell lines and report if they were recently authenticated (e.g., by STR profiling) and tested for mycoplasma contamination.	HeLa and MDA-MB-231 cells were originally obtained from American Type Culture Collection (ATCC, Manassas, USA). Stocks were frozen when received and new stocks thawed regularly to prevent genetic drift. Not recently authenicated, but are regularly checked for mycoplamsa.

* for all hyperlinks, please see the table at the top right of the document

D- Animal Models

8. Report species, strain, gender, age of animals and genetic modification status where applicable. Please detail housing and husbandry conditions and the source of animals.	NA
9. For experiments involving live vertebrates, include a statement of compliance with ethical regulations and identify the committee(s) approving the experiments.	NA
10. We recommend consulting the ARRIVE guidelines (see link list at top right) (PLoS Biol. 8(6), e1000412, 2010) to ensure that other relevant aspects of animal studies are adequately reported. See author guidelines, under 'Reporting Guidelines'. See also: NIH (see link list at top right) and MRC (see link list at top right) recommendations. Please confirm compliance.	NA

E- Human Subjects

11. Identify the committee(s) approving the study protocol.	NA
12. Include a statement confirming that informed consent was obtained from all subjects and that the experiments conformed to the principles set out in the WMA Declaration of Helsinki and the Department of Health and Human Services Belmont Report.	NA
13. For publication of patient photos, include a statement confirming that consent to publish was obtained.	NA
14. Report any restrictions on the availability (and/or on the use) of human data or samples.	NA
15. Report the clinical trial registration number (at ClinicalTrials.gov or equivalent), where applicable.	NA
16. For phase II and III randomized controlled trials, please refer to the CONSORT flow diagram (see link list at top right) and submit the CONSORT checklist (see link list at top right) with your submission. See author guidelines, under 'Reporting Guidelines'. Please confirm you have submitted this list.	NA
17. For tumor marker prognostic studies, we recommend that you follow the REMARK reporting guidelines (see link list at top right). See author guidelines, under 'Reporting Guidelines'. Please confirm you have followed these guidelines.	NA

F- Data Accessibility

18: Provide a "Data Availability" section at the end of the Materials & Methods, listing the accession codes for data generated in this study and deposited in a public database (e.g. RNA-Seq data: Gene Expression Omnibus GSE39462, Proteomics data: PRIDE PXD000208 etc.) Please refer to our author guidelines for 'Data Deposition'.<br><br>Data deposition in a public repository is mandatory for:<br>a. Protein, DNA and RNA sequences<br>b. Macromolecular structures<br>c. Crystallographic data for small molecules<br>d. Functional genomics data<br>e. Proteomics and molecular interactions	NA
19. Deposition is strongly recommended for any datasets that are central and integral to the study; please consider the journal's data policy. If no structured public repository exists for a given data type, we encourage the provision of datasets in the manuscript as a Supplementary Document (see author guidelines under 'Expanded View' or in unstructured repositories such as Dryad (see link list at top right) or Figshare (see link list at top right).	NA
20. Access to human clinical and genomic datasets should be provided with as few restrictions as possible while respecting ethical obligations to the patients and relevant medical and legal issues. If practically possible and compatible with the individual consent agreement used in the study, such data should be deposited in one of the major public access-controlled repositories such as dbGAP (see link list at top right) or EGA (see link list at top right).	NA
21. Computational models that are central and integral to a study should be shared without restrictions and provided in a machine-readable form. The relevant accession numbers or links should be provided. When possible, standardized format (SBML, CellML) should be used instead of scripts (e.g. MATLAB). Authors are strongly encouraged to follow the MIRIAM guidelines (see link list at top right) and deposit their model in a public database such as Biomodels (see link list at top right) or JWS Online (see link list at top right). If computer source code is provided with the paper, it should be deposited in a public repository or included in supplementary information.	NA

G- Dual use research of concern

22. Could your study fall under dual use research restrictions? Please check biosecurity documents (see link list at top right) and list of select agents and toxins (APHIS/CDC) (see link list at top right). According to our biosecurity guidelines, provide a statement only if it could.	NA
